# A Digital Twin Prototype for Protecting Surface Source Waters

**DOI:** 10.3390/s26196175

**Published:** 2026-09-29

**Authors:** Russell Primeau, Razak Seidu, Houxiang Zhang, Peihua Han, Guoyuan Li

**Affiliations:** Department of Ocean Operations and Civil Engineering, Norwegian University of Science and Technology in Ålesund, Postboks 1517, 6025 Ålesund, Norway; russell.b.primeau@ntnu.no (R.P.); rase@ntnu.no (R.S.); hozh@ntnu.no (H.Z.); peihua.han@ntnu.no (P.H.)

**Keywords:** water quality, digital twin, hydrodynamic model, IoT, monitoring, decision support system

## Abstract

In water safety planning, drinking water quality is protected by multiple barriers, beginning with source protection before treatment and distribution. Protection requires timely information about conditions beyond directly monitored locations. This study presents a digital twin prototype integrating networked environmental observations, a three-dimensional hydrodynamic water quality model, and source-protection applications at the Brusdalsvatnet reservoir in Norway. The framework connects state estimation for operational monitoring with contamination-scenario analysis and model-informed planning of uncrewed surface vessel sampling. Compared against observations from a single moored station throughout the 2024 season, the modelled water temperature has a root-mean-square error of 1.73 °C near the surface and 2.15 °C averaged across depth, with the largest discrepancies at intermediate depths in summer. The contaminant scenario and sampling route demonstrate analytical capabilities without field validation. The results establish a proof of concept for source-water decision support and identify remaining challenges to making its predictions reliable.

## 1. Introduction

As outlined in the World Health Organization’s Guidelines for drinking-water quality [1], water safety plans are a standard framework for ensuring the safety of drinking water supplies which has been widely and successfully adopted. Water safety planning considers the complete supply system, from catchment to consumer, with source protection providing the first barrier against contamination. For an estimated 50% of the world’s population, these sources are freshwater surface bodies [2]. Surface waters are exposed to contamination via the atmosphere and surface runoff throughout the source watershed, posing a large challenge for drinking water suppliers [3]. Trends such as urban development within watersheds and changing precipitation patterns due to climate change are projected to exacerbate the difficulty of protecting drinking water sources from contamination, increasing risks to human health [4]. The diversity and dynamism of these hazards underscore the importance of a structured, extensible approach to continuous source water monitoring which can be adapted to the specific risks and resources in each unique water supply.

Within lakes and reservoirs, water quality parameters vary spatially and temporally due to complex interactions between natural and artificial processes. These processes are impractical to control and difficult to predict. Transport of contaminants within the boundaries of a surface water body is strongly influenced by hydrodynamics, which precludes simple location-based assessment of the distribution of water quality parameters at the offtake point for a drinking water supply. Timely spatially and temporally resolved knowledge of the distribution of water quality parameters can therefore be valuable in operational monitoring, by quantifying the influence of contamination sources on influent (raw) water quality at the plant before contaminants are abstracted. However, present water quality monitoring methods typically provide low resolution and high latency in their estimate of water quality parameters, potentially failing to detect or predict contamination in time to allow effective responses before harm results [5]. The value of improving methods for detecting and monitoring contamination of surface source water bodies is widely recognized both by industry sources and in academic literature [6,7].

A digital twin combines a representation of a physical system with observations that update knowledge of its state and support analysis and decisions. Applied to source-water protection, this framework can connect routine monitoring with spatial predictions and incident-response planning, particularly for constituents that cannot be monitored continuously.

This study develops and demonstrates a systems-integration prototype for the Brusdalsvatnet drinking water reservoir in Norway. It builds on the sensing infrastructure and dashboard presented in [8] by adding a three-dimensional modelling layer and applications for state estimation, contaminant-release analysis and model-informed mobile sampling. The contribution is the integration of these components in a source-protection workflow. The governing equations, discretization and solver are established methods; their development is not a contribution of this work.

A preliminary account of this work is available as a preprint [9]; the present article supersedes it.

### 1.1. Source Water Quality

Water quality encompasses physical, chemical and biological properties that vary through a water body under the influence of sources, transport and reactions. It is infeasible to simultaneously directly monitor the distribution and trends in all water quality parameters throughout a large body of water [10]. Different hazards require different monitoring approaches. Some properties can be measured in situ using optical or electrochemical sensors, whereas many chemical contaminants, including micropollutants, and pathogenic organisms require specialized instruments or laboratory analysis [11]. Sensors suitable for continuous deployment in surface waters mainly measure physical and optical properties [12]. The spatial distributions of contaminants requiring specialized analysis are therefore generally less well observed than routine sensor variables such as temperature.

In many jurisdictions operational monitoring of public drinking water source waters via sample collection and laboratory analysis is mandatory. Digital systems have the potential to improve on the sparse, lagged, low-resolution estimates of water quality provided by this offline analysis. Automated in situ sensors at fixed locations can take measurements at high frequency for the parameters they measure. It is increasingly common for instruments to be networked for data transmission as Internet of Things (IoT) devices, providing online monitoring [13]. Transmission may use wired, Wi-Fi, broadband, or other communication mediums such as the LoRaWAN-based system presented in [14].

The spatial distribution of measurements from in situ sensors can be increased without increasing the number of sensors by locating them aboard mobile platforms such as remote-controlled or autonomous vessels capable of deploying sensors. Uncrewed surface vessels (USVs) in particular are well suited for taking in situ water quality measurements, and environmental monitoring is one of the most frequently cited applications for this class of system. However, in the review by Patterson et al. [15], such systems typically require human supervision or intervention and are not fully automatic.

Remote sensing methods such as satellite imagery are a popular alternative to automate retrieval of water quality data. Optical phenomena such as algal blooms can be monitored frequently over wide areas; for example, Qiu et al. [16] track blooms using video for frequent nearshore monitoring and satellite imagery for whole-lake coverage. However, remote sensing is less suitable than in situ methods for monitoring source water quality in many situations. Limitations include constraints on spatial, temporal and spectral resolution; susceptibility to interference from clouds and fog; and limited ability to assess many parameters essential for source water safety [17]. Therefore remote sensing methods have not been applied in this study.

To address gaps in direct measurement, it is common practice to rely on established correlations to predict the value of difficult-to-measure parameters using more easily measured indicator parameters [18]. For instance, turbidity is often used as an indicator of potential microbial contamination [19]. Data-driven approaches include soft sensors, which estimate unmeasured variables from other measurements, and surrogates, which emulate process-based model outputs without representing processes explicitly via constitutive equations [20]. Other approaches combine time-series prediction with spatial interpolation [21]. These data-driven approaches require suitable data for training and evaluation.

Process-based hydrodynamic water quality models (HWQMs) represent transport and reactions explicitly using conservation laws coupled to reaction formulations, allowing them to be used when observations are sparse or the event of interest has not occurred. Their capabilities and limitations have been studied extensively [22,23]. Process-based models are more explainable, but not inherently more accurate [24]. Their predictions still depend on uncertain boundary conditions, initial conditions, parameters and process formulations. This paper uses this established modelling approach as a component of the integrated system, rather than attempting to establish its general validity.

Three-dimensional resolution preserves variation in both horizontal directions and depth, supporting comparisons between sampling locations and the examination of subsurface plume pathways. One-dimensional models can reproduce vertical stratification, the layering of a water column by temperature and density, and laterally averaged two-dimensional models can represent depth-dependent transport; the required spatial detail depends on the application [25]. The three-dimensional representation was selected to support the spatial analyses in Section 3. Its site-specific performance is assessed separately from the established numerical method.

At Brusdalsvatnet, ammonium and nitrate data are limited to sparse laboratory analyses of grab samples, many at or below the detection limit. The release considered in this case study was hypothetical and did not occur, so no observations of the resulting plume exist. A process-based model provides a way to examine the scenario, but the quantitative water-quality evaluation presented here is limited to temperature; validation against chemical and biological observations remains future work.

### 1.2. Digital Twins

Digital twin frameworks connect observations of a physical system with an updated virtual representation and functions for monitoring, prediction and analysis [26,27]. For source-water protection, these functions correspond to estimating present conditions, exploring contamination scenarios and planning monitoring actions. An online dashboard provides an interface through which water managers can inspect observations and model results.

Digital twins are used across engineered and natural systems, with communication requirements and useful update intervals determined by the application [28,29]. In the water sector they support treatment and distribution operations [30,31]. For environmental monitoring, the one-to-three-hour latency described by Murphy et al. [32] provides a practical near-real-time target; whether an integrated modelling system meets it requires measurement of the full observation-to-publication interval.

Recently, several works [33,34,35] have identified digital twins as a practical approach for monitoring and managing complex natural systems. Strict definitions of digital twin such as that of Kritzinger et al. [36], which require control in the form of automatic data flow from the virtual object to the real object, would preclude this framing. Clearly, it is impractical to control the state of many natural systems, such as the temperature of a lake spanning several kilometres. Conversely, there are well-established models of natural systems with data assimilation which meet less restrictive definitions of digital twins. For instance, Blair [37] suggests considering operational weather forecasting systems as digital twins of the atmosphere, arguing that it is useful to apply this re-framing to identify possible innovations through knowledge transfer from methods developed for monitoring and control of artificial systems. Digital twins are therefore understood to include systems which lack automated control of the real object within this paper, in keeping with the usage established by these works. What such systems offer in place of actuation is decision support. Hazeleger et al. [38] describe digital twins of the Earth as decision support systems for environmental challenges, whose purpose is to monitor, forecast and assess the system together with the consequences of human intervention in it; within the water sector, Brahmbhatt et al. [39] couple a twin of a distribution network to a decision support system for quality regulation and leak localization. The prototype described here is of that kind. It estimates a state that is not observed directly, and every action that follows is taken by an operator.

Research on this topic is nascent, with several ongoing projects having published preliminary results. Vasilijevic et al. [40] present a system for monitoring and simulating numerous parameters representing the state of water in a coastal area. Xu et al. [41] present a twin of an urban river system, incorporating a variety of data sources, and Yao et al. [42] present a system with similar applications in predicting water quality in an urban river system from real-time sensors. Qiu et al. [16] describe a digital twin lake framework built around water quality and harmful algal blooms, integrating multi-source monitoring data for visualization and analysis. At the catchment and sector scale, Li et al. [43] survey digital twin applications across water conservancy infrastructure, and Kwon et al. [44] review hybrid physical and soft-sensor architectures for river digital twins. Reported systems of this kind are therefore no longer rare. However, to the authors’ knowledge, very few have been reported for surface drinking water reservoirs specifically, and most of the examples above derive their state estimate from interpolation, data-driven surrogates, or visualization of sensor networks rather than from a three-dimensional process-based model. Operational three-dimensional lake platforms do exist: Baracchini et al. [45] maintain continuously updated hydrodynamic forecasts for several lakes, supported by an automated calibration framework [46]. What is claimed as novel here is accordingly not the model, the equations or the solver, all of which are standard, but the systems integration: a near real-time in situ sensing network coupled to a three-dimensional process-based model of a drinking water source, and the operational applications that this coupling makes possible—a nowcast, an estimate of conditions as they are rather than a forecast of conditions to come, for operational monitoring; hazard assessment by simulation, and model-spread-informed planning of mobile sampling.

### 1.3. Framework for Source Water Quality Digital Twins

**Figure 1:**
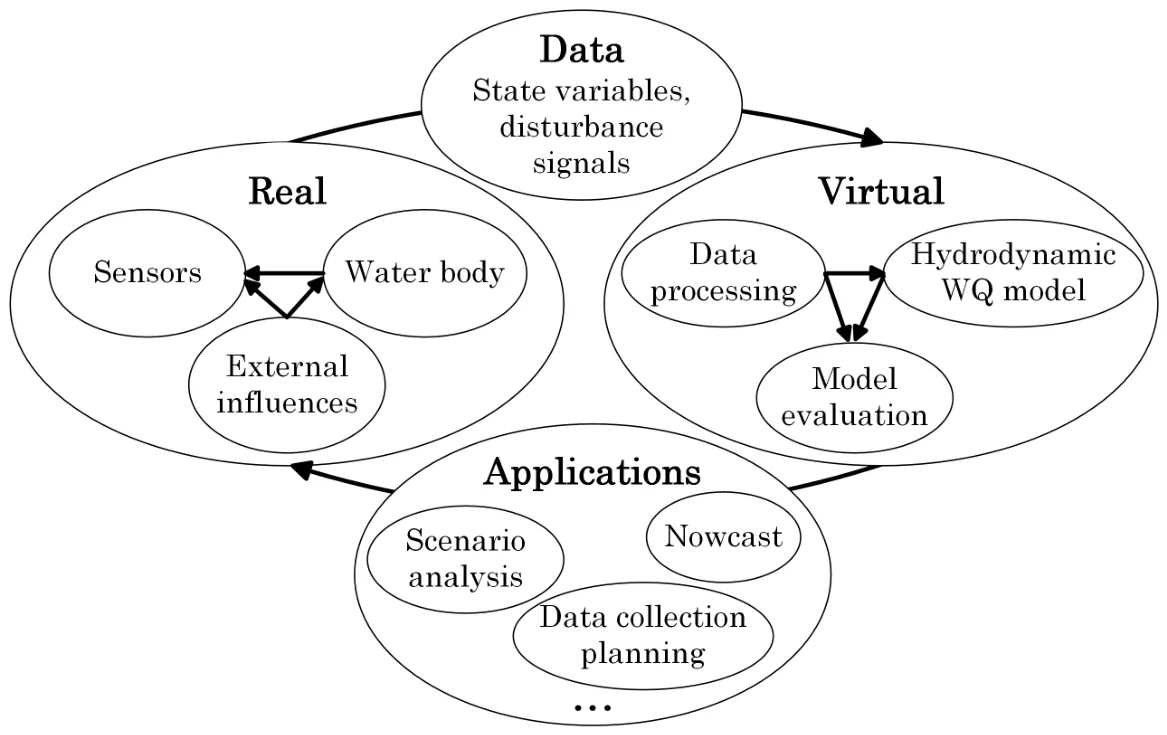
Digital twin framework adapted for surface water quality.

The features of digital twins identified in Section 1.2 are suitable for the application of source water quality monitoring as summarized in Section 1.1, subject to the availability of appropriate and compatible components. A framework for such an integration adapted from Grieves’ generic digital twin concept is presented in Figure 1 [47]. The proposed water quality digital twin consists of a system of hardware and software components to enable communication between the real object and its virtual counterpart.

The water body itself constitutes the real object which the digital twin duplicates. Its state is influenced by disturbance signals from external phenomena such as weather and contamination events. IoT sensors collect data on the state of the real object and the disturbance signals, described further in Section 2.1. These data are transmitted in the real-to-virtual direction via telecommunications networks to the virtual components of the system. There, certain processed data are used as input to calculate updates to a water quality model, while other data are used to evaluate and validate the model outputs.

The model outputs support decision-making by water-quality managers, who may use this information to guide monitoring and management actions in the physical system. Thus, the virtual-to-real pathway is mediated by human decision-making rather than automated actuation. The applications presented here include monitoring through a calibrated model nowcast, scenario analysis for contamination events, and planning of USV data-collection missions.

## 2. Materials and Methods

### 2.1. Sensing Systems

**Figure 2:**
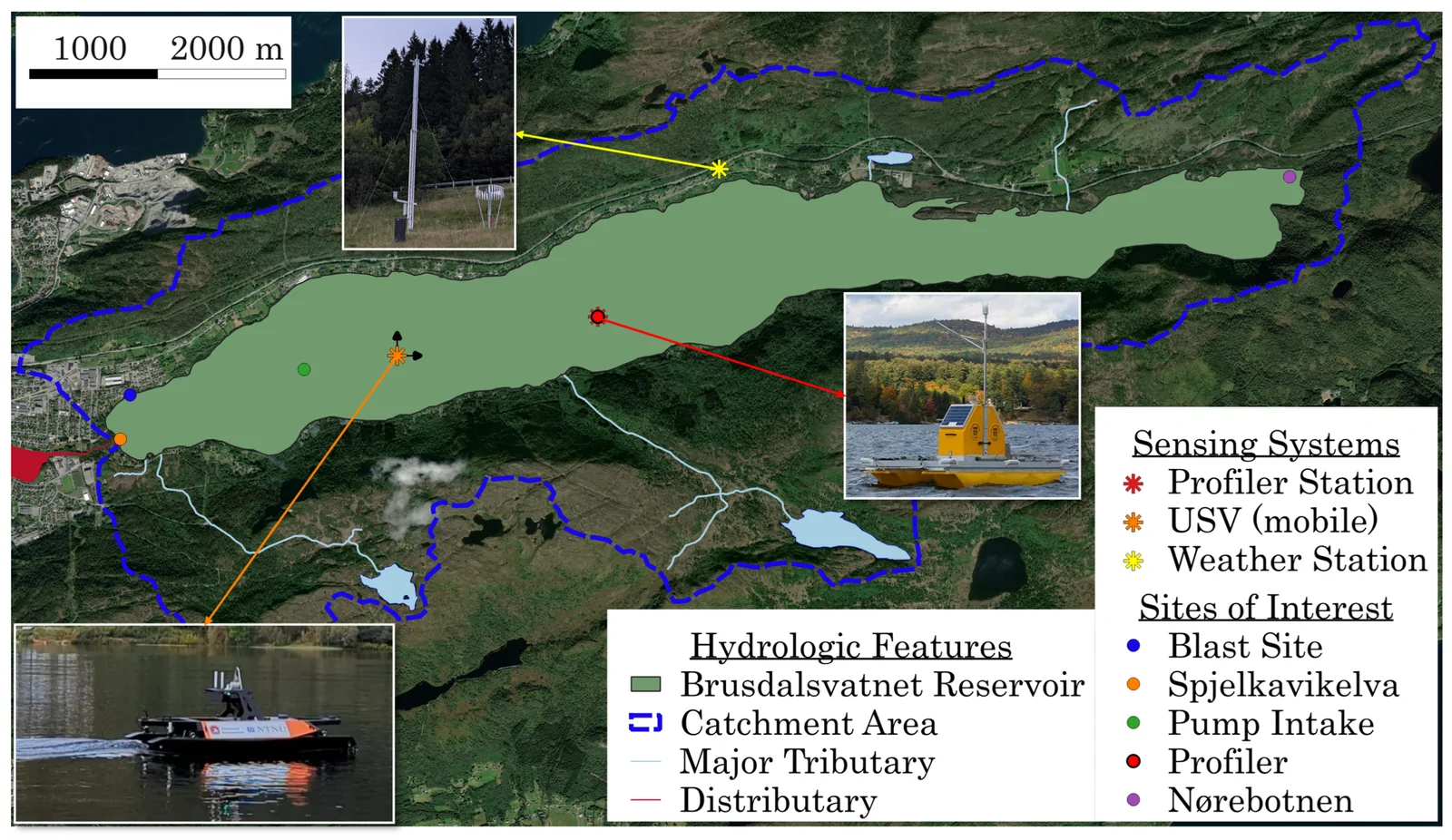
Brusdalsvatnet catchment area, showing the sensing platforms of Section 2.1 and the five sites of interest analysed in Section 3.2, including the release point of the hypothetical scenario considered there; no such release has occurred. Web Mercator projection (EPSG:3857), centred at 62.4723° N, 6.4774° E; base imagery ESRI World Imagery (Clarity).

In the case study, three types of IoT sensing platforms are used to collect data on real conditions: a land-based weather station, a moored floating profiler station, and a USV. Locations and images of these systems are depicted in Figure 2.

- Weather station: Parameters measured by the weather station include shortwave radiation (W/$m^{2}$), precipitation (mm), wind direction (°), wind speed (m/s), air temperature (°C) and relative humidity, which are used to define boundary conditions in the HWQM. The weather station includes several independent mast-mounted instruments, integrated with data storage and a wireless modem for broadband internet connectivity. The weather data are logged locally and backed up on a web server hosted by the manufacturer, where it is accessed by the digital twin script. The on-shore system’s location near the north shore of the lake was selected for weather conditions representative of Brusdalsvatnet’s entire area.
- Profiler: A Vertical Profiler System developed by YSI Integrated Services & Systems provides in situ measurement of the 8 water quality parameters listed in Table A1 of Appendix A, together with the operational calibration record for each channel. The system consists of a moored floating platform, which provides power and a broadband (4G) datalink for a multiparameter sonde and datalogger. A depth-sensor-equipped EXO sonde, also by YSI Integrated Services & Systems, is mounted on a winched cable. Every 12 h the sonde is lowered to collect a vertical profile, logging each parameter at approximately 1 m intervals down to a depth of 50 m. The same parameters are also logged hourly from the sonde’s resting position near the surface at 2.95 m depth.
  The profiling platform has a fixed horizontal position in 80 m of water. To prevent damage from lake ice, the platform is removed from the water for approximately five to six months per year. The active season typically runs from April to November. In situ water quality sensors must be recalibrated regularly to correct for sensor drift, which can cause noteworthy errors in the time series data.
- USV: An Otter USV by Maritime Robotics AS equipped with an instrument sonde provides the ability to take measurements from remote locations semi-automatically at lower cost and effort than with manual methods. The Otter includes water quality sensors mounted in an AML-3 sonde provided by AML Oceanographic, which can be configured to measure water temperature, conductivity, pH, turbidity, or other parameters. Like the profiler station, the sonde is mounted to a winched cable to allow the measurement of vertical profiles. The USV contains a GPS antenna for position sensing, which is used for navigation and geotagging recorded data. Thus the USV’s data collection capabilities in principle include four degrees of freedom (DOFs): the x,y position of the USV on the surface of the water, the depth *z* of the sonde in the water, and the time *t* at which measurements are taken. Each DOF is constrained, for instance by the 3D extents of the water body and the speed of the USV.
  Automation of USV operations is limited by manual processes for launch, recovery, and data extraction. The manufacturer specifies an endurance of approximately 20 h at 2 kn [48], so the practical limit on a mission is the duration a supervised deployment can occupy rather than the vessel’s range. Instead of continuous monitoring, USV data collection is conducted in discrete missions.

Additional information about the specification and calibration of the sensors is presented in Appendix A, including links to public documentation from the manufacturers.

### 2.2. Hydrodynamic Water Quality Model

**Figure 3:**
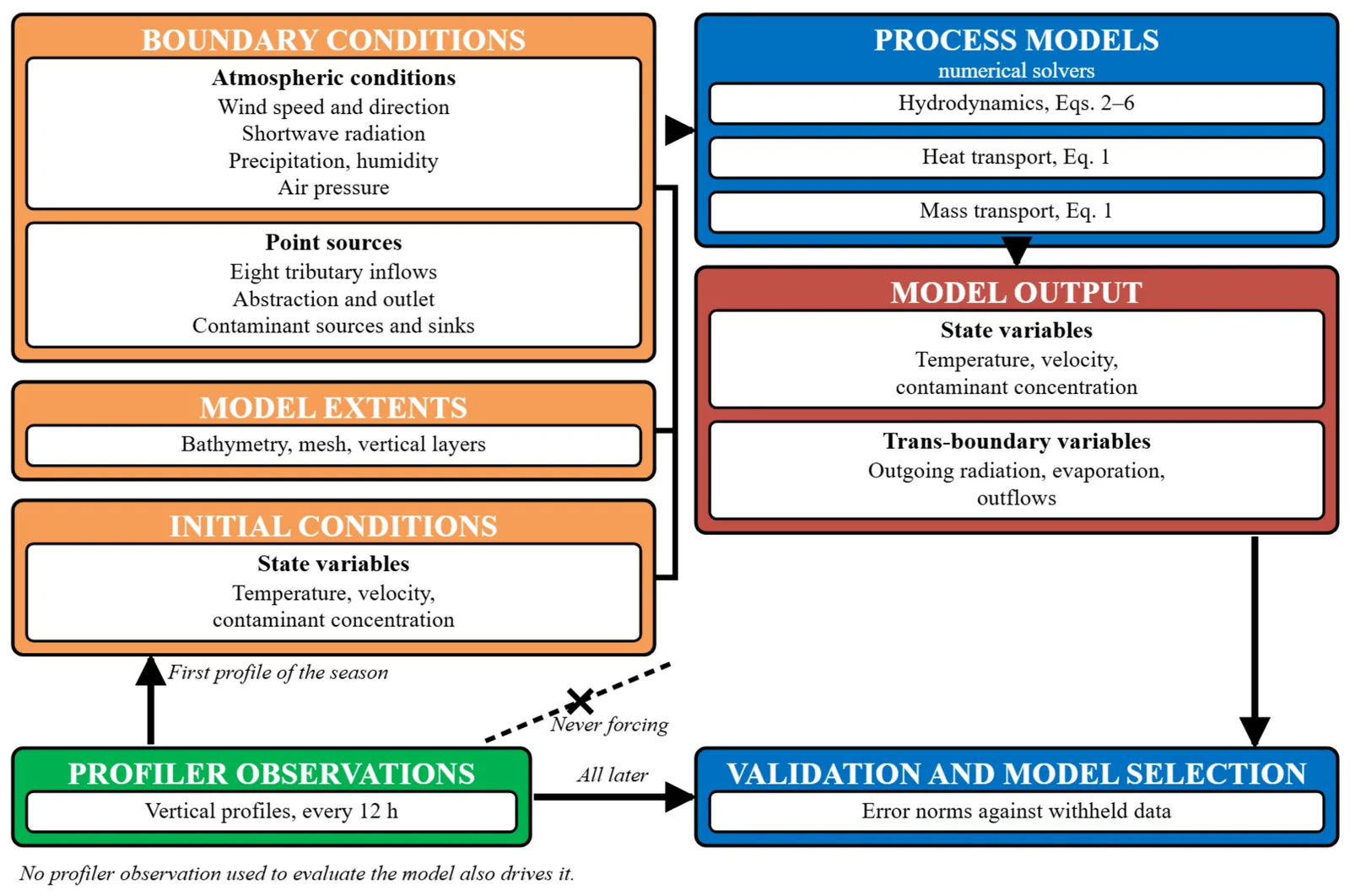
Inputs, solvers and outputs of the HWQM. The initial profiler observation informs the initial temperature field; subsequent profiles are compared with model output rather than directly prescribed as temperatures within the simulated water column. Equation numbers refer to Section 2.2. Colour denotes role here and in Figure 4: green for instruments, orange for measured data and the conditions derived from it, blue for solvers and analysis, brick for model output, gold for decision products.

The HWQM combines sensor data and prior knowledge to estimate the state of the water body. This input–output relationship is summarized in Figure 3. Boundary condition data are defined at every model timestep using data from the sensing platforms. Each channel of sensor data requires error checking for unrealistic values due to causes such as calibration drift, low voltages, communication disruptions, or other anomalies. One practical limit on the model update frequency is the sample rate of these sensing platforms, modulated by the bandwidth of communications systems. For boundary conditions which are not measured by any sensing system, values may be estimated from external modelling or prior knowledge. Initial conditions which span the physical extent of the model might be derived from measurements, but likewise require prior knowledge or assumptions about the hydrodynamic system. In an operational model with continuous, regular updates, the initial conditions may be defined using output from a previously computed timestep. Outputs can be organized into state variables which span the physical extent and time range of the model run, as well as process variables which describe how the hydrodynamic system interacts with the environment outside its boundaries.

The model configuration is assessed offline before use in an operational update loop. The configuration comparison described in Section 3.1 comprises twenty-one configurations generated by iterative adjustment across successive runs rather than by an automated search, counting once a configuration that was run to more than one end date. Ten cover the full season, and together they span the ranges reported in Appendix B: horizontal mesh resolution, vertical layer count, water clarity, horizontal and vertical filtering, the vertical eddy coefficients, the Courant limit and the maximum timestep. Their retained results provide the cost–accuracy comparison in Section 3.1. The thermal simulation evaluated in Section 3.1 is a separate configuration, documented in Appendix B; it is not one of the points plotted in that comparison. It was selected separately using agreement in local water-column heat content at the profiler station rather than the pooled depth-band RMSE used in that comparison. The contaminant and mobile-sampling examples use outputs from a separate scenario configuration. These experiments demonstrate complementary parts of the framework and do not constitute a single set of simulations using one configuration.

The process-model module in Figure 3 uses the Delft3D FM hydrodynamic and water quality solvers. The following equations summarize the standard hydrostatic formulation; discretization, closure and numerical solution are documented by Deltares [22,49]. For an incompressible flow, constituent concentration $C_{n}$ obeys the advection–diffusion–reaction balance

(1) $$\frac{\partial C_{n}}{\partial t}=\nabla \;\cdot \left(D\nabla C_{n}\right)-u\;\cdot \nabla C_{n}+\sum S_{n}$$

where $C_{n}$ has units of mass per volume, u=(u,v,w) is velocity, D is the dispersion tensor, and $S_{n}$ denotes source and sink rates per unit volume. The divergence form allows spatially varying dispersion coefficients. Heat transport uses the corresponding energy balance with surface heat-flux terms.

For the prismatic control volume defined by each cell on the 2D surface mesh, the water depth *h* can be solved using the continuity equation in Equation (2):

(2) $$\frac{\partial h}{\partial t}=\int_{0}^{h}\left(q_{\mathrm{in}}-q_{\mathrm{out}}\right)dz+P_{\mathrm{precip}}-E_{\mathrm{evap}}-\frac{\partial (\bar{u}h)}{\partial x}-\frac{\partial (\bar{v}h)}{\partial y}$$

where $q_{\mathrm{in}}$, $q_{\mathrm{out}}$ are local inflows and outflows as volume flow rates normalized by total cell volume, with dimensions of inverse time; $P_{\mathrm{precip}}$ is the rate of precipitation; $E_{\mathrm{evap}}$ is the rate of evaporation; and $\bar{u}$, $\bar{v}$ are the depth-averaged horizontal velocity components.

For each 3D cell in the mesh, velocity components in the *x*- and *y*-direction can be solved for using the horizontal momentum equations, Equations (3) and (4) respectively:

(3) $$\frac{\partial u}{\partial t}=-u\frac{\partial u}{\partial x}-v\frac{\partial u}{\partial y}-w\frac{\partial u}{\partial z}+fv-\frac{1}{\rho }\frac{\partial P}{\partial x}+F_{x}+\frac{\partial }{\partial z}\left(\nu_{V}\frac{\partial u}{\partial z}\right)+M_{x}$$

(4) $$\frac{\partial v}{\partial t}=-u\frac{\partial v}{\partial x}-v\frac{\partial v}{\partial y}-w\frac{\partial v}{\partial z}-fu-\frac{1}{\rho }\frac{\partial P}{\partial y}+F_{y}+\frac{\partial }{\partial z}\left(\nu_{V}\frac{\partial v}{\partial z}\right)+M_{y}$$

where ρ is mass density; *P* is pressure; *f* is the Coriolis parameter, representing the apparent force due to the Earth’s rotation at a particular latitude; $\nu_{V}$ is the vertical eddy viscosity coefficient; $F_{x}$, $F_{y}$ represent horizontal stress-divergence contributions per unit mass; $M_{x}$, $M_{y}$ represent other momentum-source contributions. Each has dimensions of acceleration.

Under the hydrostatic approximation, vertical acceleration is neglected in the momentum balance. For incompressible flow away from volumetric sources, continuity gives Equation (5):

(5) $$\frac{\partial w}{\partial z}=-\left(\frac{\partial u}{\partial x}+\frac{\partial v}{\partial y}\right)$$

The momentum equations use Reynolds averaging, with unresolved turbulent transport represented by closure and eddy coefficients. The thermal configuration uses the *k*-ϵ closure; its settings are given in Appendix B.

The hydrostatic pressure relationship is given in Equation (6):

(6) $$\frac{\partial P}{\partial z}=-\rho g$$

Surface heat exchange, wind stress, bed friction, inflows, outflows and constituent reactions complete the forcing of this system. These established processes are configured for the case study rather than introduced as new numerical methods.

The first profiler observation informs the initial temperature field. Subsequent profiles are used to compare simulated and observed conditions rather than directly prescribing temperatures in interior cells. Figure 3 makes this distinction explicit.

**Figure 4:**
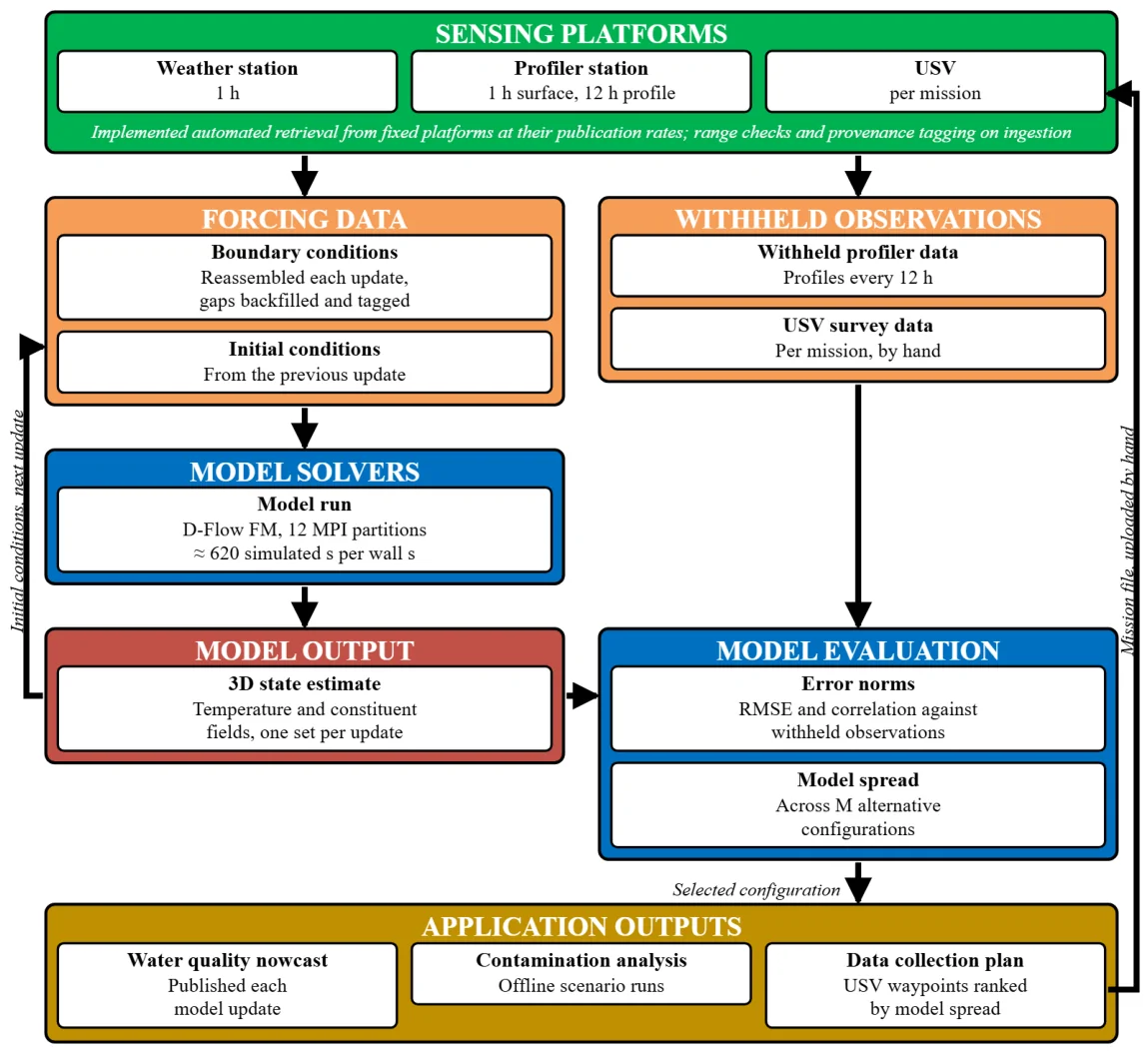
Proposed operational update loop assembled from the prototype components. Surface-sonde and weather retrieval are hourly and vertical profiles are retrieved twice daily; vessel data transfer is manual. Model restart, selection and publication illustrate the intended integration rather than a demonstrated unattended loop. Implementation status, data coverage and solver throughput are reported in Appendix F.

Figure 4 shows the intended operational architecture. Newly retrieved observations would update model boundary conditions; model output would supply a spatial state estimate and the initial conditions for a later update. Alternative configurations could be compared against available observations before selecting an output for publication. This proposed operational ensemble is distinct from both the configuration comparison of Section 3.1 and the transport ensemble used for sampling-plan analysis in Appendix E.

The implemented components support data retrieval, input preparation, model execution, evaluation and display, but the full sequence has not been demonstrated as an unattended hourly loop. Scenario analysis and sampling-plan generation are demonstrated offline. Appendix F distinguishes implemented components from proposed automation and reports sensor coverage and measured solver throughput.

Python scripts and Windows Task Scheduler support the implemented workflow. A local application provides access to model preparation and analysis, while the public dashboard described in [8] displays monitoring data and selected outputs. Table 1 reports the computing environment for the thermal-run throughput benchmark.

The acquisition scripts retrieve surface-sonde and weather observations hourly and vertical profiles twice daily. Offline comparison against the profiler record provides a check on the resulting thermal field. Evaluating both stratified and cooling periods exposes errors that may be less apparent over a short spring window; it does not by itself identify the process responsible for a discrepancy.

Delft3D FM was selected for its established hydrodynamic and water quality capabilities and prior applications to seasonally stratified lakes [50]. Solver settings for the thermal simulation are recorded in Appendix B. The case study covers the open-water season: ice processes are not represented in this configuration, and the profiler is removed during winter to prevent damage.

### 2.3. Data Collection Optimization

One recommended component of emergency response plans is increased monitoring, for which USVs provide useful capabilities. However, the four degrees of freedom in each measurement taken by the USV sonde create an operational requirement which does not exist for fixed platforms: the location, sequence and timing of data collection must be planned. This is an analytical task for which the digital twin framework is well-suited, with bidirectional flow of information allowing prior sensor inputs and simulated system dynamics to inform the collection of new data.

The fixed profiler samples a single horizontal location. Mobile profiles can instead target locations where alternative model predictions differ, providing observations with which to compare those alternatives. Because each mission has limited time for transit and profiling, selecting a small set of such locations is a useful planning task.

To maximize the informativeness of the data collected by this system, an automated method for planning individual data collection missions based on the locations with the greatest model-spread scores is implemented in the digital twin system. Although each measurement is characterized by four coordinates—x,y,z,t—the present planner optimizes only the horizontal waypoint coordinates x,y. Depth profiles are collected according to a predefined procedure, and measurement timing is determined by route execution. Given the complexity of other practical constraints of launching and recovering the USV, the method is limited to optimizing the location and order of data collection points, without considering the time at which measurements will be taken. The shorter the interval between the model state used for planning and execution of the mission, the more informative the collected data are expected to be.

Alternative simulations are generated using different input or process assumptions. For horizontal cell *i* of the *I* cells in the horizontal mesh, with $L_{i}$ active layers, write $P_{i\ell }^{\left(m\right)}$ for the concentration predicted in layer *ℓ* by alternative *m*. The score is the layer mean of the relative divergence among the *M* alternatives, evaluated where there is a predicted signal to measure:

(7) $$\begin{array}{cc} \mu_{i\ell } & =\frac{1}{M}\sum_{m=1}^{M}P_{i\ell }^{\left(m\right)},\;s_{i\ell }=\sqrt{\frac{1}{M}\sum_{m=1}^{M}{\left(P_{i\ell }^{\left(m\right)}-\mu_{i\ell }\right)}^{2}},\;{\bar{P}}_{i}=\frac{1}{L_{i}}\sum_{\ell =1}^{L_{i}}\mu_{i\ell },\\ L_{i}^{+} & =\left\{\ell \in \left\{1,\ldots ,L_{i}\right\}:\mu_{i\ell }>0\right\},\;L_{i}^{+}=\left|L_{i}^{+}\right|,\\ \sigma_{i} & =\frac{1}{L_{i}^{+}}\sum_{\ell \in L_{i}^{+}}\frac{s_{i\ell }}{\mu_{i\ell }}\;\mathrm{for}\;i\in D,\;D=\left\{\;i\in \left\{1,\ldots ,I\right\}\;:\;{\bar{P}}_{i}\geq \tau \max_{j\in \{1,\ldots ,I\}}{\bar{P}}_{j}\;\right\} \end{array}$$

In Equation (7), $s_{i\ell }$ is the standard deviation across alternatives in layer *ℓ* and $\mu_{i\ell }$ their mean, so their ratio is the divergence in that layer relative to the quantity predicted there, and ${\bar{P}}_{i}$ is the layer-mean prediction at the cell. The layer mean is arithmetic, not a thickness-weighted depth average. The set $L_{i}^{+}$ contains the layers with a positive ensemble mean. In a zero-mean layer, all non-negative tracer predictions are zero, so both $s_{i\ell }$ and $\mu_{i\ell }$ are zero and their ratio is undefined; such layers are omitted rather than assigned an artificial zero contribution. The denominator $L_{i}^{+}$ is therefore the number of layers included in the score, matching the implementation. Ranking cells by the absolute divergence $s_{i\ell }$ would rank them by how much of the released material they hold, because that divergence scales with the quantity present; the ratio instead identifies where the alternatives disagree about whether the release has arrived at all.

The ratio becomes unstable as a positive $\mu_{i\ell }$ approaches zero, so only the cells in D are ranked: those whose layer-mean predicted concentration reaches a fraction τ of the largest anywhere in the mesh. This model-signal eligibility threshold limits the influence of large relative differences where the predicted quantity is negligible; it is not an analytical or instrument detection limit. A value of $\tau =10^{-3}$ is used here. At the horizon reported in Section 3.3, it leaves half the mesh eligible, excluding the far field beyond roughly 3.7 km from the release, which the plume has not reached.

The score measures how far the alternatives diverge from one another at a given location, and is used only to rank locations against each other. The alternatives used here differ in the hydrodynamic solution that carries the released material, so the score measures sensitivity of the predicted distribution to the choice of flow configuration. Alternatives that shared one flow solution and varied only the reaction applied to the transported material would answer a different sensitivity question. Their divergence would be a pointwise function of the concentration field and would tend to emphasize the immediate neighborhood of the release, rather than differences in where the hydrodynamic configurations carry it. The score has no calibrated meaning: it orders locations, and carries no information about the precision or accuracy of any individual prediction. This article uses spreadfor this quantity throughout, reserving dispersion for the physical transport process of Section 2.2.

Scores are normalized as $\sigma_{i}^{\prime }=\sigma_{i}/\max_{j\in D}\sigma_{j}$ where that maximum is positive. Division by a single positive constant is monotone, so the normalization leaves the ranking of cells, the relative cluster weights and the selected route unchanged; its purpose is to make reported weights comparable between runs. In the retained analysis the highest-scoring cells were identified before normalization and the constant is the maximum over that retained set, which is the same value because the retained set contains the global maximum. The *r* highest-scoring cells are selected in Equation (8) as

(8) $$R^{*}=\underset{\begin{array}{c} S\subset \{1,\ldots ,I\},\\ |S|=r \end{array}}{\mathrm{argmax}}\sum_{i\in S}\sigma_{i}^{\prime }$$

To avoid redundant measurements within a single area with high model-spread scores, density-based spatial clustering of applications with noise (DBSCAN) groups cells lying within a threshold distance of one another, and returns a shorter set of coordinates consisting of the centroids of these groups. With a minimum of one cell per group, the procedure is single-linkage at that threshold, so the threshold is the separation two groups must have rather than the width one group may reach: a chain of cells each within the threshold of the next forms a single group whatever distance the chain spans. Writing $c_{k}$ for the set of cells assigned to cluster *k*, each of the *K* clusters receives a weight $w_{k}$ equal to the sum of the normalized scores of its member cells, as given in Equation (9):

(9) $$w_{k}=\sum_{j\in c_{k}}\sigma_{j}^{\prime },\;\mathrm{for}\;k=1,\ldots ,K$$

Write $p_{k}$ for the centroid of cluster *k* and $p_{0}$ for a specified launch-and-recovery point, and let d(·,·) denote geodesic distance. The ordered sequence of distinct cluster centroids with maximum summed weight under a route-length budget $D_{\max}$, as given in Equation (10), is found exactly by dynamic programming over the set of clusters already visited and the last one visited. Distances include travel from and back to the launch point:

(10) $$C^{*}=\underset{\begin{array}{c} (k_{1},\ldots ,k_{q})\;\mathrm{distinct},\;1\leq q\leq K\\ d\left(p_{0},p_{k_{1}}\right)+\sum_{j=1}^{q-1}d\left(p_{k_{j}},p_{k_{j+1}}\right)+d\left(p_{k_{q}},p_{0}\right)\leq D_{\max} \end{array}}{\mathrm{argmax}}\sum_{j=1}^{q}w_{k_{j}}$$

If there are multiple sets with equal total weights, the set with the shortest total distance is chosen. This list of coordinates $C^{*}$ can be exported in the file format used to define a remotely operated data collection mission for the USV, with data collection at each waypoint.

### 2.4. Case Study Area

The case study integrated the monitoring equipment and models according to the framework described in Section 2 to create a twin of the Brusdalsvatnet reservoir located in Ålesund Municipality, Norway. The lake is nutrient-poor and mixes from top to bottom twice a year, in spring and autumn. This enlarged natural lake is the main drinking water source for two municipalities with a combined population of nearly 70,000. The main basin is seasonally stratified; the fixed profiler provides detailed vertical observations at one horizontal location. Basic physical characteristics of the lake are presented in Table 2.

Physical features, sensing locations and the five scenario-analysis sites are mapped in Figure 2. Hydrological boundary conditions are externally supplied time series of tributary discharge, outlet flow and water abstraction, applied at eight tributaries and the two principal withdrawals. Their generation is outside the scope of this integration study. The thermal simulation uses time-varying supplied forcing; the scenario outputs in Section 3.2 were generated with a different model and forcing configuration.

Third-party lake-level and outflow records contribute to the provenance of the hydrological inputs. Comparison of the thermal simulation against the available water-level observations gives a root-mean-square error (RMSE) of 0.027 m and Pearson correlation 0.987 over 3180 paired values, after aligning the level datum; these are the values reported in the water-level row of Table 3. They describe agreement of that simulation with the monitoring record. Those records cannot be redistributed, as the Data Availability Statement notes.

Previous assessment has identified several specific hazards to Brusdalsvatnet’s water quality. Historically, non-point pollution from stormwater runoff has carried microbial contaminants from animal activity within the watershed into the lake. Roadway expansion and traffic growth have been linked to heavy metal contamination. Point sources of pollution such as leakages from aging wastewater infrastructure in the urbanized western end of the watershed have been linked to increased *E. coli* concentrations in [51]. Conversely, the limited nutrient inflows and high latitude provide strong inherent protection against eutrophication, the nutrient enrichment that drives algal growth, limiting the need to measure chlorophyll *a* or other related parameters.

## 3. Results

### 3.1. Water Quality Nowcast

**Figure 5:**
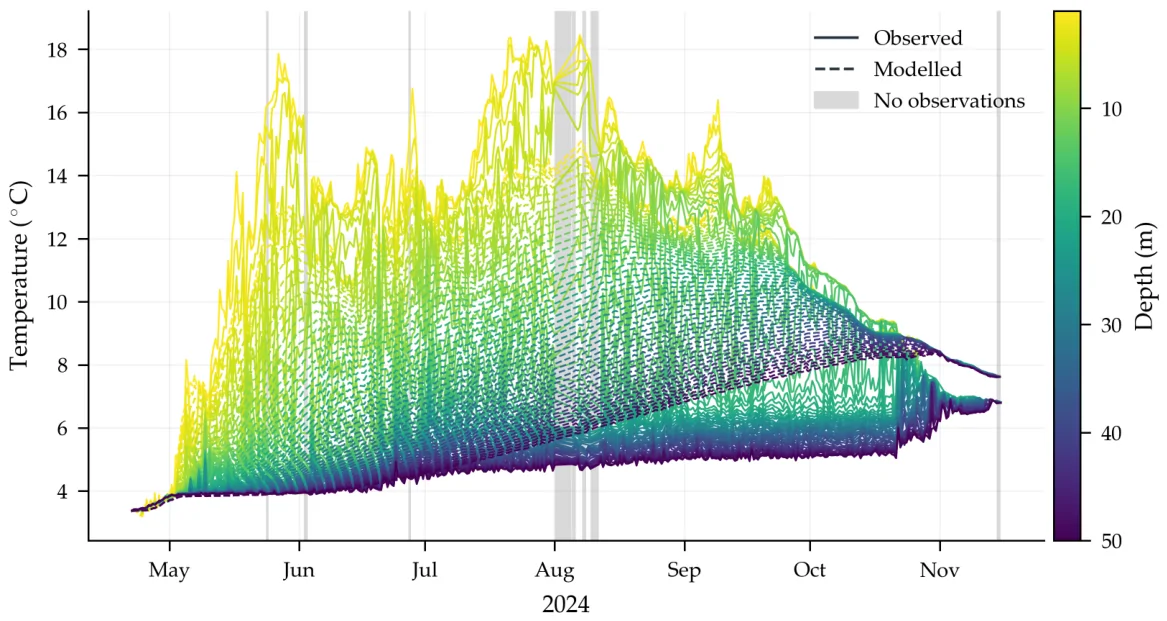
Observed (solid) and modelled (dashed) water temperature at the profiler station for the thermal simulation, 22 April to 20 November 2024, in fifty one-metre depth groups; colour denotes depth. Root-mean-square error is 1.73 °C at 2.95 m and 2.15 °C averaged across the depth bands. Shaded bands indicate periods without observations. The same paired records underlie Table 3 and Appendix C.

The skill of a model configuration is evaluated by comparing its vertical temperature profile against the observed time series of vertical temperature profiles from the profiler station. Figure 5 shows that comparison for the configuration evaluated in Section 3.1, across all fifty depth groups. As Xia et al. [52] summarize, calibration of 3D hydrodynamic models to temperature alone can overlook important flow dynamics, yet remains common because temperature profiles are comparatively inexpensive to obtain and are routinely included in lake monitoring. Velocity observations require additional instruments distributed across locations and depths, are seldom available, and turn calibration into a multisite, multivariable problem. Temperature is therefore the most practical calibration variable for this study and provides information about several interacting physical processes, but the absence of velocity observations limits what the resulting agreement establishes about transport. Here temperature provides the largest reliable and non-static observational dataset available for computing error terms. Surveys of lake modelling practice likewise describe calibration as contending with limited direct measurements [53]. Assessments of aquatic ecosystem models find that validation routinely stops at the comparison of simulated state variables against observations, with the process rates and fluxes that would constrain circulation seldom tested [54]. The left and right extremities of the series in Figure 5 also illustrate how these twice-yearly mixing events leave the water column at a uniform temperature, providing well-defined initial and final conditions.

**Figure 6:**
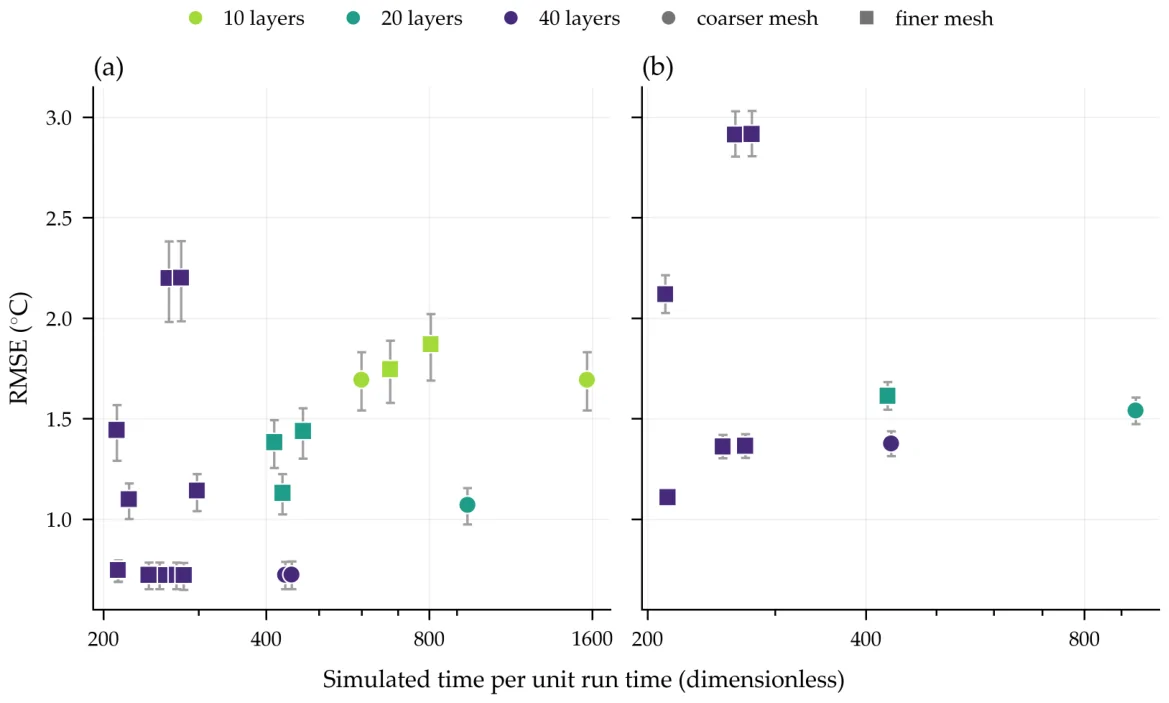
Model accuracy and computational cost for the configuration comparison. Markers denote configurations; colour indicates vertical layers and symbol horizontal mesh. Temperature RMSE is pooled across one-metre depth bands; computational performance is simulated time divided by wall-clock time (dimensionless), with larger values indicating faster runs. Bars are marginal 95% day-block-bootstrap intervals, not pairwise comparisons. (**a**) All 21 configurations over their common period, 25 April–27 June 2024; (**b**) the 10 configurations extending to 15 November 2024. Values are comparable within, but not between, panels. The configurations plotted here are distinct from the thermal simulation reported in Table 3, which was selected using water-column heat content at the profiler station.

Figure 6 summarizes the exploratory model-configuration comparison and its computational cost. The shorter runs cover the coarser part of the comparison, whereas the seasonal runs cover the more refined settings. All runs in this comparison were executed serially on one workstation, so their throughput is not comparable with the partitioned benchmark in Appendix F. Resampling whole days preserves residual correlation within a day.

Over the full season (Figure 6b), refining the horizontal mesh at 40 layers from 36,197 to 65,900 three-dimensional cells changes RMSE from 1.377 to 1.365 °C for a 1.6-fold increase in run time, while changing the remaining settings at that resolution spans 1.110 to 2.916 °C. Physical settings therefore have a larger effect on this observation-based comparison than the tested refinement, which is why the mesh was fixed and the effort directed at the physical parameters. Across the search as a whole (Figure 6a), the six lowest RMSE values have substantially overlapping descriptive intervals while differing twofold in cost. Because these are marginal intervals rather than paired comparisons, the overlap does not establish equivalence; it shows that the observed RMSE differences are small relative to the within-configuration resampling variability. This is a limited resolution and configuration comparison, not proof of grid-independent transport, and the thermal simulation evaluated below is a separate configuration, so the figure does not describe how that configuration was reached.

For context, Feldbauer et al. [55] compared four calibrated one-dimensional lake-temperature models across 73 lakes. The best-performing model for each lake gave median RMSE 1.2 °C and median correlation 0.98, with 95% below 2 °C. Direct comparison is limited by differences in observation coverage, dimensionality and error aggregation.

Table 3 reports the thermal simulation over 22 April–20 November 2024. Agreement varies with depth, and not monotonically: RMSE reaches its minimum of 1.12 °C at 7 m and its maximum of 3.07 °C at 18 m, then declines steadily to 1.57 °C at 50 m. Twenty-three of the fifty depth bands have RMSE below 2 °C. The final-error column records model minus observation at the last available paired time at each depth; it is not mean seasonal bias. Figure 5 plots the same paired records across all depth groups, and the profile and residual plots in Appendix C show excessive warming below the surface during stratification.

The full-season comparison exposes errors that a short spring window would not characterize. The model captures broad seasonal thermal evolution, but the stratified-period discrepancies limit quantitative interpretation of subsurface temperatures and associated transport.

### 3.2. Hazard Assessment

**Figure 7:**
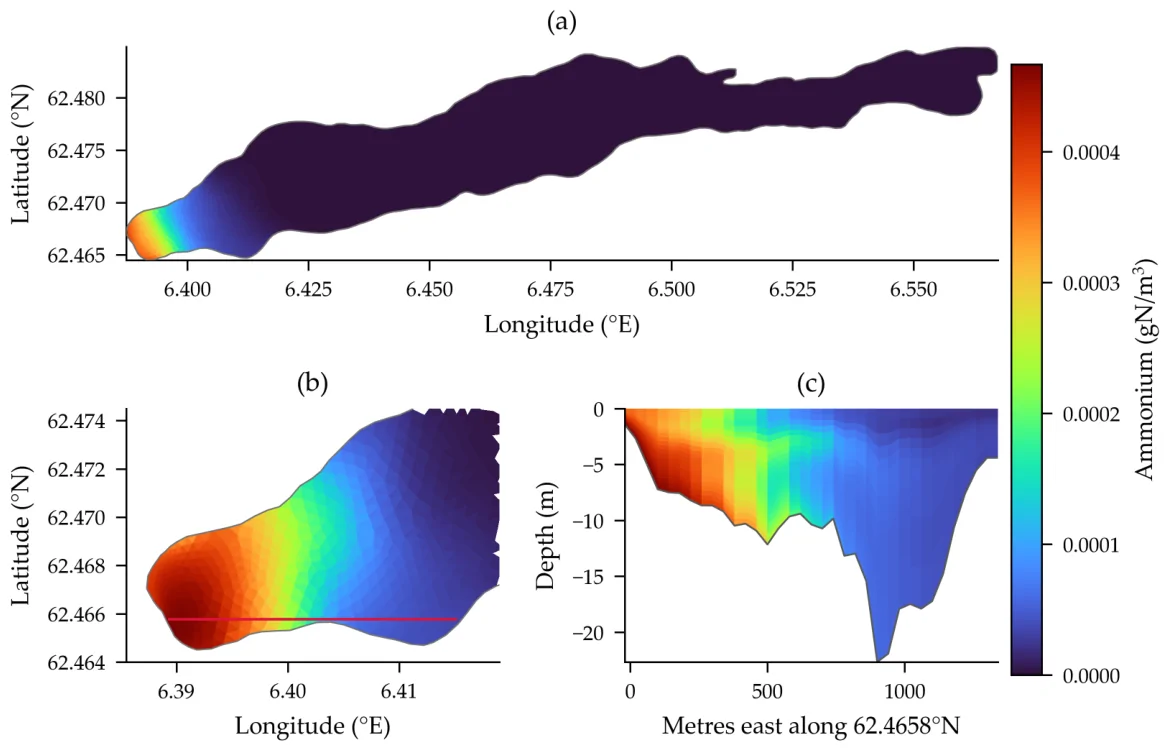
Modelled ammonium concentration in gN/$m^{3}$ 36 h after the hypothetical release: (**a**) in the surface layer, across the whole reservoir; (**b**) in the layer immediately above the bed, over the western basin where the release occurs; and (**c**) a vertical section eastwards along the horizontal red line at 62.4658° N marked in (**b**). The vertical grid divides the water column into twenty layers of equal fraction, so a layer follows the bed rather than lying at a fixed depth; the section in (**c**) is drawn between the true layer boundaries. All three panels share one colour scale, so concentrations may be compared between them. Output of the scenario simulation, which uses a different configuration from the thermal simulation of Table 3. No release occurred, and these concentrations have not been compared against measurements.

**Figure 8:**
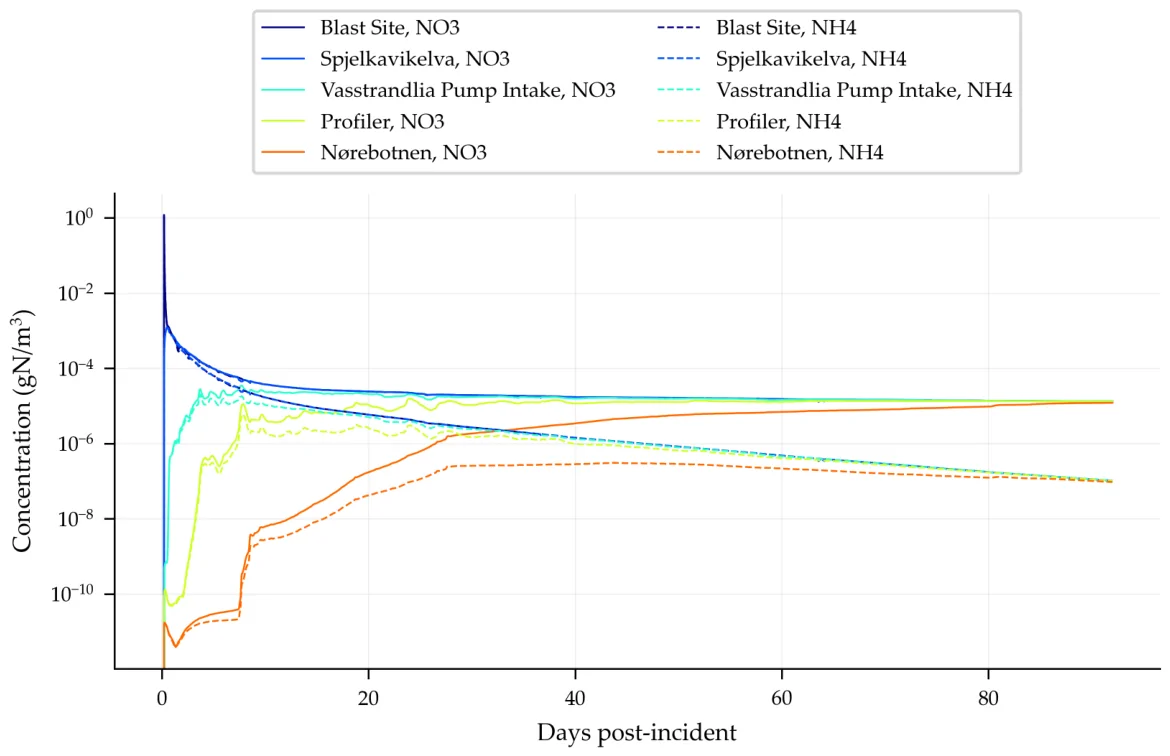
Scenario simulation output: depth-averaged ammonium and nitrate concentrations at five sites, in gN/$m^{3}$, versus time after release. Lines show model output, not measured concentrations, and give the increment above ambient rather than the total concentration.

A configured HWQM can be used offline to examine the transport consequences of a hypothetical contaminant release. Construction blasting can introduce ammonium nitrate residues to the lake over a short period. The scenario was motivated by a contractor’s risk analysis for a construction project in the catchment. The implemented source was 3.6 kg of nitrogen. No comparable release was observed at the site; the scenario demonstrates how the model could support consequence assessment and response planning. Figure 7 and Figure 8 show outputs from a separate scenario configuration, not the thermal simulation in Table 3. The sparse laboratory nitrogen samples cannot resolve a transient plume, so these outputs demonstrate the analysis workflow rather than validated concentration forecasts.

The implemented source, 3.6 kg of nitrogen, is equivalent to approximately 10.3 kg of ammonium nitrate (${\mathrm{NH}}_{4}$${\mathrm{NO}}_{3}$), which is 35% nitrogen by mass. Dissociation gives equal molar amounts of ammonium and nitrate, and each ion carries one nitrogen atom, so the nitrogen divides equally between the two species irrespective of their differing molar masses: 1.8 kg as ammonium-N and 1.8 kg as nitrate-N. The simulations represent this load as a constant rate of 0.5 gN/s in each species over one hour. Concentrations are reported as the increment above ambient, in gN/$m^{3}$; the simulations start from zero and no claim is made here about background concentrations in the lake.

The site of the initial release is identified in Figure 2 as “Blast Site,” along with four other sites where water quality is of interest: the intake for the pump which supplies the Vasstrandlia water treatment plant, the outlet where the reservoir drains into the river Spjelkavikelva, the profiler station at which observational data are collected, and Nørebotnen to represent the far-field dispersion. The intake abstracts at approximately 35 m depth. Contaminants are simulated as being released between 04:00 and 05:00 on 1 August 2024.

Both nitrogen species are chemically reactive and thus, in addition to transport, their concentrations are affected by reaction processes. Nitrification and denitrification reaction process models are included in the simulation scenario presented here. The overall stoichiometry of nitrification and denitrification is presented in Equations (11) and (12), with organic matter represented as ${\mathrm{CH}}_{2}$O in the latter, since denitrification in the water column proceeds heterotrophically with organic carbon as the electron donor.

(11) $${\mathrm{NH}}_{4}^{+}+2O_{2}\to {\mathrm{NO}}_{3}^{-}+2H^{+}+H_{2}O$$

(12) $$5{\mathrm{CH}}_{2}O+4{\mathrm{NO}}_{3}^{-}+4H^{+}\to 2N_{2}+5{\mathrm{CO}}_{2}+7H_{2}O$$

These overall reactions are not solved directly. They are implemented in the process library as first-order rate expressions modulated by temperature and by dissolved oxygen concentration, following the nitrification and denitrification process formulations documented in [49]; the resulting rates enter Equation (1) as *S* terms for the respective chemical species.

The scenario simulation uses meteorological forcing assembled for that experiment, with the hydrodynamic solution beginning in spring so that stratification is established before the release. It is computed on the 4935-cell horizontal mesh with twenty sigma layers. The release occurs on 1 August 2024, and the water-quality calculation is coupled to the hydrodynamics at an hourly interval from that moment to 1 November 2024. This window falls inside the period covered by the thermal simulation, but the two are separate runs on separate configurations and their outputs are not interchangeable. Coverage statistics for the forcing used in the thermal simulation are reported in Appendix F; they are not the provenance of the scenario runs.

The simulated dispersal of the contaminant plume 36 h after release is depicted in Figure 7. Figure 7a shows the surface layer, where the release remains confined to the western end of the reservoir. Figure 7b shows the layer immediately above the bed over that same area, and the two panels share a colour scale: the model places the highest concentration at the bed rather than at the surface, despite the release entering the surface layer. The section in Figure 7c, eastwards along the line marked in Figure 7b, shows why. The released material mixes downward as it travels, and the vertical contrast grows with distance: at the western end of the section the bed concentration exceeds the surface value by about a quarter, and between 500 and 700 m it is roughly twice the surface value. Over that stretch, where the bed lies within about 13 m of the surface, the maximum sits in the lowest layer. Beyond about 700 m the maximum leaves the bed, which falls away to 20 m and more, and travels instead at a depth of 3 to 7 m with more dilute water both above and below; the column maximum remains 1.7 to 1.9 times the surface concentration out to the eastern end of the section. This example highlights the value of representing three-dimensional dispersion, which could be poorly resolved by lower-resolution models or missed by sparse point measurements. In this case, there are implications for planning interventions. Within this simulated scenario, surface-only containment measures such as floating booms would not intercept the modelled subsurface maximum; this is an inference from the simulation rather than an experimentally demonstrated finding, and it would need to be confirmed against measurements before being relied upon operationally.

To visualize the timing of the plume’s dispersal, depth-averaged concentration of nitrate and ammonium at the five sites of interest identified in Figure 2 are plotted against time in Figure 8. Predicting the timing and duration of elevated concentrations is important for quantifying the expected harm, which in turn is necessary for assessing cost-benefit tradeoffs of interventions. In this case, the model produces a large short-term spike in the concentrations of both nitrogen species at the blast site and nearby Spjelkavikelva, but concentrations at the remaining sites are only marginally elevated before the simulated increments decay toward zero. Ammonium concentration decays more quickly as it is more affected by the reaction processes, while nitrate concentrations are more persistent.

Routine grab samples provide scale context: the retained in-lake records span 0.001–0.011 gN/$m^{3}$ ammonium and 0.079–0.120 gN/$m^{3}$ nitrate. These observations concern background conditions, not the hypothetical plume, and do not establish an analytical detection threshold for an increment above background.

These outputs show a strongly localized early peak and smaller depth-averaged concentrations at the other sites. A depth average at the intake location does not give the concentration at the abstraction depth. Neither these outputs nor background variability establish that an increment would be undetectable or that mitigation is unnecessary. Appendix D reports the whole-domain balance for a matched conservative-tracer transport calculation, which closes to 14 parts per million of the applied load, and Appendix E reports the observed sensitivity of predicted concentrations across the tested discretizations.

For a decision about mitigation, the source loading would need to be constrained by incident-specific evidence, with predictions at the abstraction depth and an assessment of relevant uncertainties. The present demonstration shows how spatial and temporal model output can organize that analysis and identify monitoring locations; it does not demonstrate that an intervention would work.

### 3.3. Optimized Data Collection Plan

Increased monitoring is a recommended component of incident response plans for identified hazards. To demonstrate the planning method of Section 2.3, the release of Section 3.2 was repeated on a set of alternative hydrodynamic solutions, described in Appendix E. Each member applies the same nitrogen load at the same cell and hour and carries it as a conservative tracer, so that they differ in how the release is carried and not in how it reacts; they differ in the vertical mixing of the flow solver, and one of them in whether the vertical filter is active.

**Figure 9:**
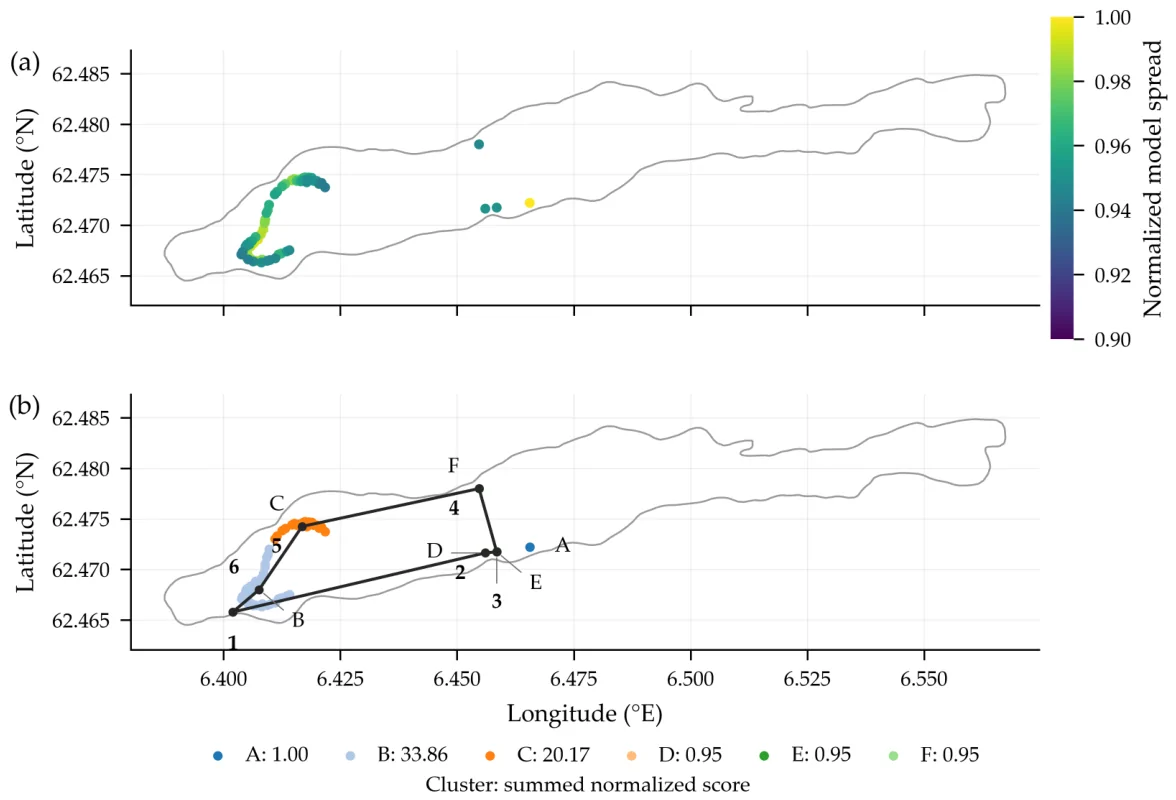
Data collection planning from the retained 60-cell dataset: (**a**) normalized model-spread scores at the retained cells; (**b**) the 60 cells grouped into clusters, their normalized cluster weights, and the selected 6942 m route. Number 1 marks the launch-and-recovery point; numbers 2–6 mark visits to clusters D, E, F, C and B, respectively. Dot colours in (**a**) represent normalized model spread; in (**b**) they identify clusters A–F, with summed normalized scores given in the legend. Black markers and lines indicate the selected waypoints and route. Scores derive from five alternative hydrodynamic solutions carrying the same release, so they measure sensitivity of the predicted distribution to the configuration of the flow solver. The route was not executed. Cluster labels and weights are generated from the same dataset as the optimization.

The score is evaluated on the daily output at 00:00 on 7 August 2024, 5.8 days after the release. That horizon is a planning assumption representing approximately when a survey mounted after a reported incident could be in the water. The retained set depends on this choice: over the thirty simulated days the 60 highest-scoring cells form between one and seven clusters and span between 0.45 and 4.4 km from east to west. The score field over the retained cells is shown in Figure 9a.

The retained set of 60 highest-scoring cells is clustered using DBSCAN with a 100 m neighborhood and a minimum of one cell per cluster. The neighborhood is a pragmatic mission-design setting informed by the spatial continuity of the score field: its empirical semivariogram reaches a quarter of its sill at 138 m, half at 262 m and the sill itself at about 790 m. A 100 m radius therefore groups nearby high-scoring cells while limiting redundant visits. It does not show that a profile at one cell represents another, or determine an optimal sampling density. The resulting six clusters are labelled A–F, and each centroid is assigned the sum of its member scores. Two of them hold 35 and 21 cells and span 648 and 560 m along their long axes, because the neighborhood sets how far apart two clusters must be, not how far a single cluster may extend; the other four are single cells. Every centroid falls within 20 m of a modelled cell, so each waypoint lies in open water. The retained cells lie between 89 and 438 m from the shoreline, with a median of 230 m, so reaching them calls for a vessel rather than bank access. Varying the neighborhood between 40 and 300 m changes the number of groups from 21 to four and the number of visited stations from 19 to three. Over that range the summed score of the selected route moves by under 2% and its length between 6484 and 6942 m, although the centroids and route geometry also change. The neighborhood therefore primarily controls how finely the mission is divided in this demonstration; Appendix G reports the sweep.

Which cells are retained depends on the ensemble as a whole. Taking every subset of four of the five members in turn, a mean of 54% of the 60 cells is shared with the set the full ensemble produces, ranging from 0 to 75% across subsets; over every subset of three the mean is 23%. The retained cells should therefore be read as the output of this ensemble rather than as individually robust locations, and a larger ensemble would be needed before they could be read otherwise.

The distance budget is 7 km, a limit on the duration of a supervised mission rather than on the vessel’s endurance. At 2 kn it allows approximately 1.9 h of transit; profiling, launch and recovery require additional time. The selected route visits D, E, F, C and B before returning to the launch point, travelling 6942 m. It maximizes the summed cluster score and then minimizes distance under the stated assumptions. Because the budget binds, the optimizer has to leave something out rather than simply order the visits: Cluster A lies 3359 m from the launch point and carries a normalized weight of 1.00 against 33.86 for B and 20.17 for C, so the detour to reach it does not repay itself. Below approximately 6.25 km the route reaches at most three clusters, and at 7.5 km and above it reaches all six, at which point no selection remains to be made. Shoreline obstacles, profiling duration and time-varying information value are not optimized.

Appendix G examines sensitivity of the downstream clustering and selected geographical regions to resampling the retained cells.

This data collection plan is exported as a mission file in the format the USV accepts. The route shown in Figure 9b is an executable mission generated by the prototype; it was not executed, and no field data were collected against it. Executing such a mission would require uploading the file to the USV, which must be manually launched and recovered before the collected data are uploaded to the digital twin’s database. As shown in the model update loop in Figure 4, data from a USV mission would then be used to calculate a new error norm from each alternative model that made predictions for times compatible with the time of collection, to compare the accuracy of the alternative models.

## 4. Discussion

The prototype connects monitoring observations with spatial modelling and source-protection analyses. Its practical contribution is the ability to use a common information workflow for inspecting conditions, exploring scenarios and identifying mobile sampling locations. It supports the information needs of a water safety plan; it does not replace treatment barriers, management procedures or the water supplier’s assessment of an incident.

The thermal comparison gives RMSE 1.73 °C against the hourly record at 2.95 m, with Pearson correlation 0.962 over 4504 paired values, and a mean of 2.15 °C across the fifty depth bands (Table 3). It therefore demonstrates agreement in broad seasonal evolution alongside substantial discrepancies below the surface during stratification. Temperature agreement alone leaves the velocity field that drives constituent transport largely unconstrained: different combinations of mixing, surface exchange and forcing can produce similar temperatures while producing different circulation [52]. Evaluation of state variables, process rates and system behaviour provides complementary evidence [54]. Water-current measurements or tracer observations would strengthen the transport assessment, but are not available for this demonstration. The comparison is also confined to a single moored station, so the horizontal structure of the modelled fields, the very thing the framework is built to provide, remains unconstrained by observation.

The nitrogen scenario and the sampling plan are conditional demonstrations. The source is a hypothetical worst-case scenario motivated by a contractor’s risk analysis rather than a measured release, and no observation of such a release exists against which the predicted plume could be checked. The transport ensemble provides a model-spread score, not a complete uncertainty characterization. Cell resampling tests the downstream planning algorithm but cannot establish robustness to additional model alternatives. Field comparison with alternative sampling strategies is needed to determine whether the proposed planning method improves information gained per mission.

Sensor gaps and the distinction between implemented components and an unattended service also limit operational maturity. Appendix F reports data coverage and solver throughput. Publication latency, recovery from failures and clear indication of stale output require end-to-end testing before operational reliance.

## 5. Conclusions

This study demonstrates a digital twin prototype integrating networked observations, an established three-dimensional model and applications for source-water protection. The contribution is the systems integration and its use for state estimation, hypothetical-release analysis and mobile-sampling planning. The numerical method and solver are established components.

For the thermal simulation, hourly temperature at 2.95 m agrees with observations at RMSE 1.73 °C and Pearson correlation 0.962; the mean depth-band RMSE is 2.15 °C, with the largest errors at intermediate depths. These comparisons are all at one moored station, and no observation constrains the modelled horizontal field. Chemical predictions and the sampling strategy remain unvalidated demonstrations, and the complete unattended operational loop has not been established. These limits distinguish the capabilities of the framework from the evidence provided by this case study.

Developing and calibrating the three-dimensional process-based model required substantial observations and computation. Its value lies in its explicit representation of transport and reactions and its resolution of three-dimensional structure. Applications that do not require those capabilities may be better served by simpler approaches.

The framework is potentially transferable, subject to local bathymetry, forcing, hazards, instrumentation and institutional capacity [56]. Further work should address:

- Validation of the modelled chemical and biological constituents against a tracer experiment or a monitored release.
- Independent validation of the velocity field, from water-current measurements or drifters, so that transport results do not rest on a temperature fit alone.
- Extension of the refinement study of Appendix E across independently generated meshes and with replication. Across the two meshes and two vertical resolutions tested here, peak depth-averaged concentration changes by median values of 6 to 16%; establishing grid independence requires additional refinement and a convergence analysis.
- Separation of the observational record into distinct calibration and validation periods, and enlargement of the transport ensemble beyond five members, since Section 3.3 shows the retained cells to be sensitive to which members are included.
- Field execution of a planned mission, and comparison of the resulting information gain against uniform, random and expert-chosen sampling.
- End-to-end operation of the update loop as an unattended service, including measured publication latency, behaviour under retrieval and simulation failures, and alerting that distinguishes a stale nowcast from a current one.
- Replication at water bodies differing in size, depth, residence time and dominant hazard, to establish the conditions under which the architecture provides useful decision support.

## Figures and Tables

**Table 1:** Computing environment for the thermal simulation and for the throughput benchmark in Appendix F. The model-configuration comparison reported in Section 3.1 was run serially on a separate machine.

Component	Specification
CPU	2× Intel Xeon Gold 5118 (2.30 GHz)
Memory	32 GB RAM
Operating System	Windows 10 Education
Code Execution	Python 3.11

**Table 2:** Brusdalsvatnet characteristics.

Property	Value	Units
Maximum depth	102	m
Surface Area	7.495	km^2^
Catchment Area	27.766	km^2^
Volume	0.3	km^3^

**Table 3:** Thermal simulation performance, 2024 season. MAE denotes mean absolute error; final error is model minus observation at the last paired time. The depth-band mean row reports the mean sample count and mean statistics across the fifty bands.

Comparison	N	Pearson r	RMSE	MAE	Final Error
Water temperature, hourly at 2.95 m	4504	0.962	1.73 °C	1.40 °C	+0.79 °C
Water temperature, profile at 1 m	353	0.959	1.94 °C	1.59 °C	+0.32 °C
Water temperature, profile at 20 m	389	0.819	2.95 °C	2.53 °C	+0.80 °C
Water temperature, profile at 30 m	395	0.865	2.42 °C	2.00 °C	+0.81 °C
Water temperature, profile at 50 m	398	0.875	1.57 °C	1.14 °C	+0.82 °C
Water temperature, mean of 50 depth bands	380	0.867	2.15 °C	1.75 °C	—
Water level	3180	0.987	0.027 m	0.018 m	−0.050 m

**Figure A1:**
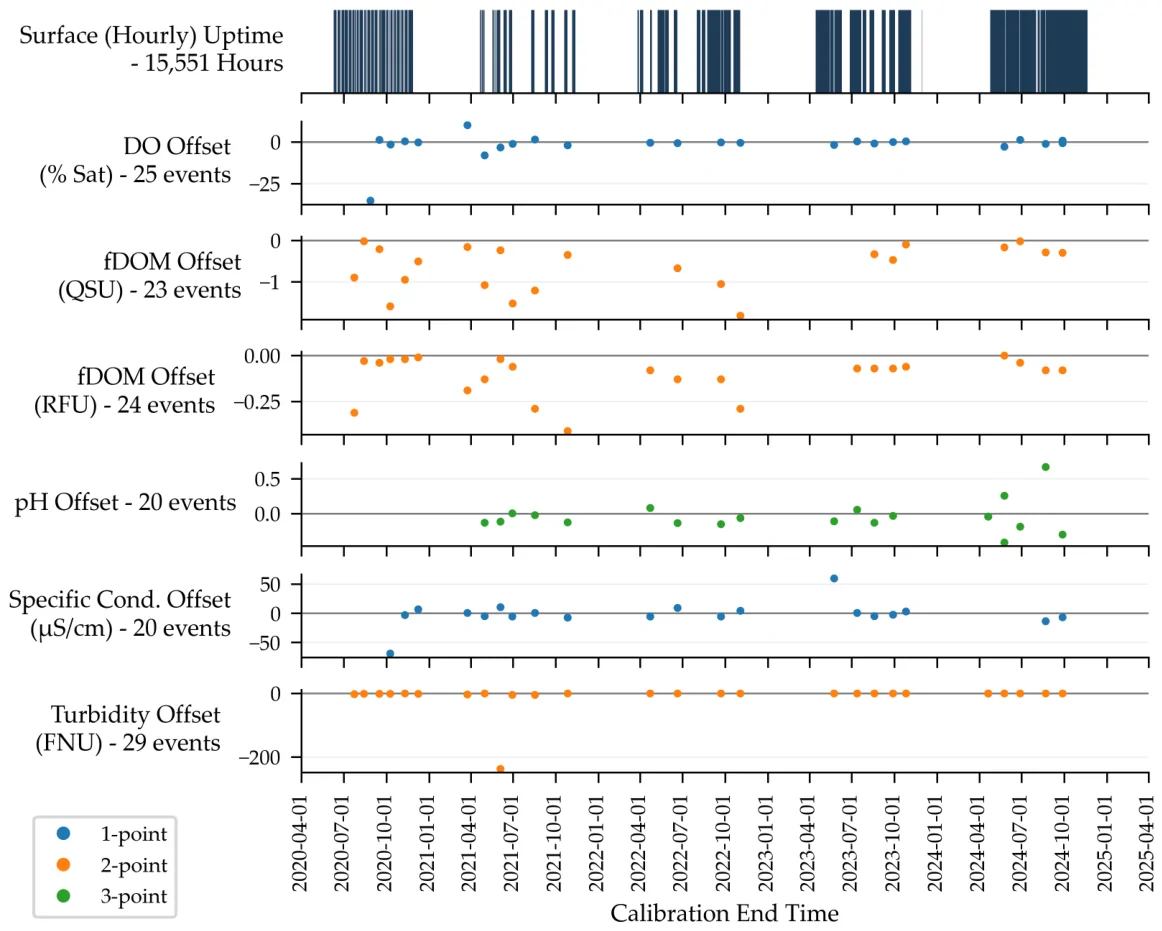
Profiler calibration history.

**Figure A2:**
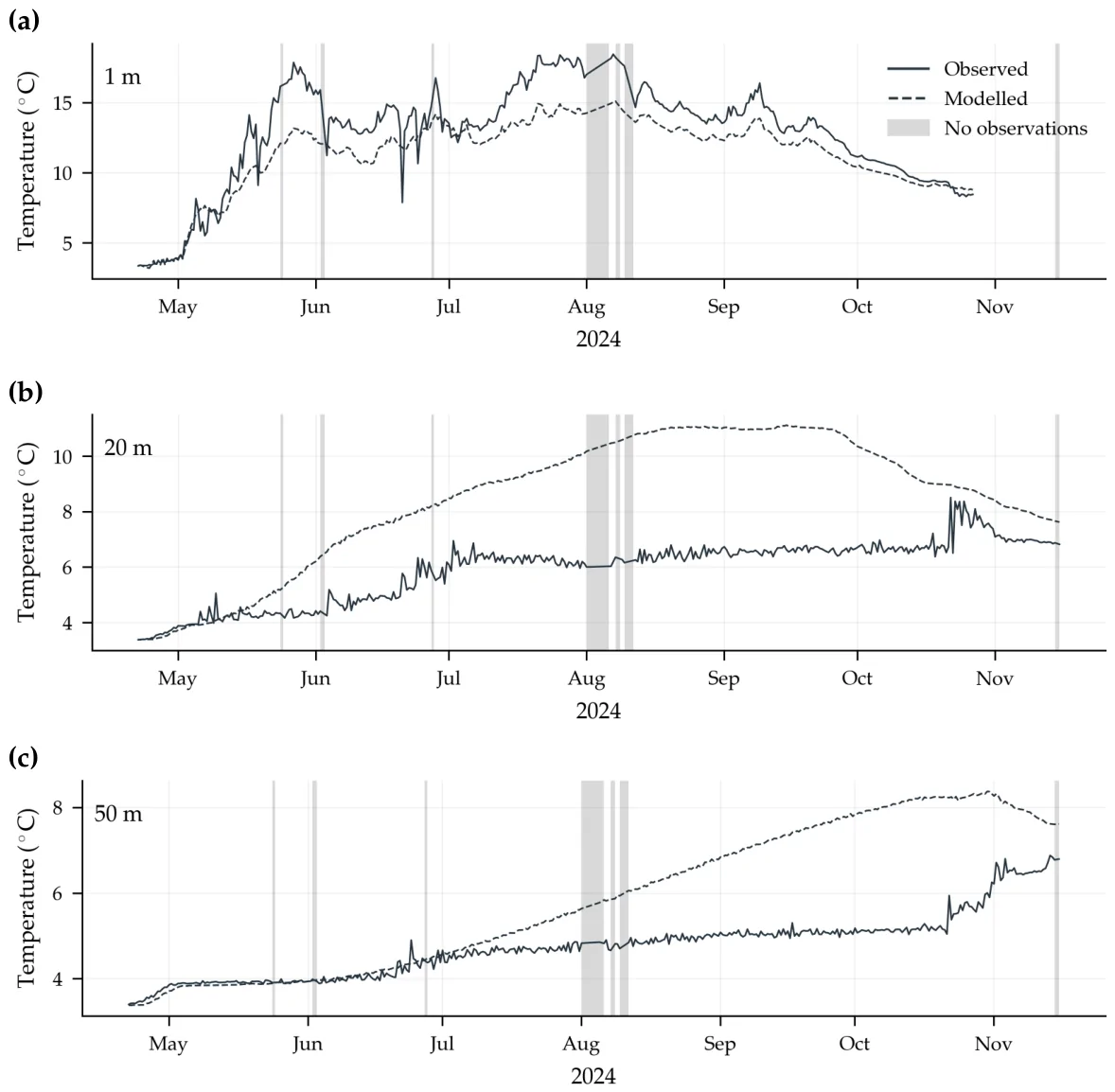
Observed (solid) and modelled (dashed) water temperature at the profiler station for the thermal simulation, (**a**) at 1 m, (**b**) at 20 m, and (**c**) at 50 m. Shaded bands indicate periods without observations.

**Figure A3:**
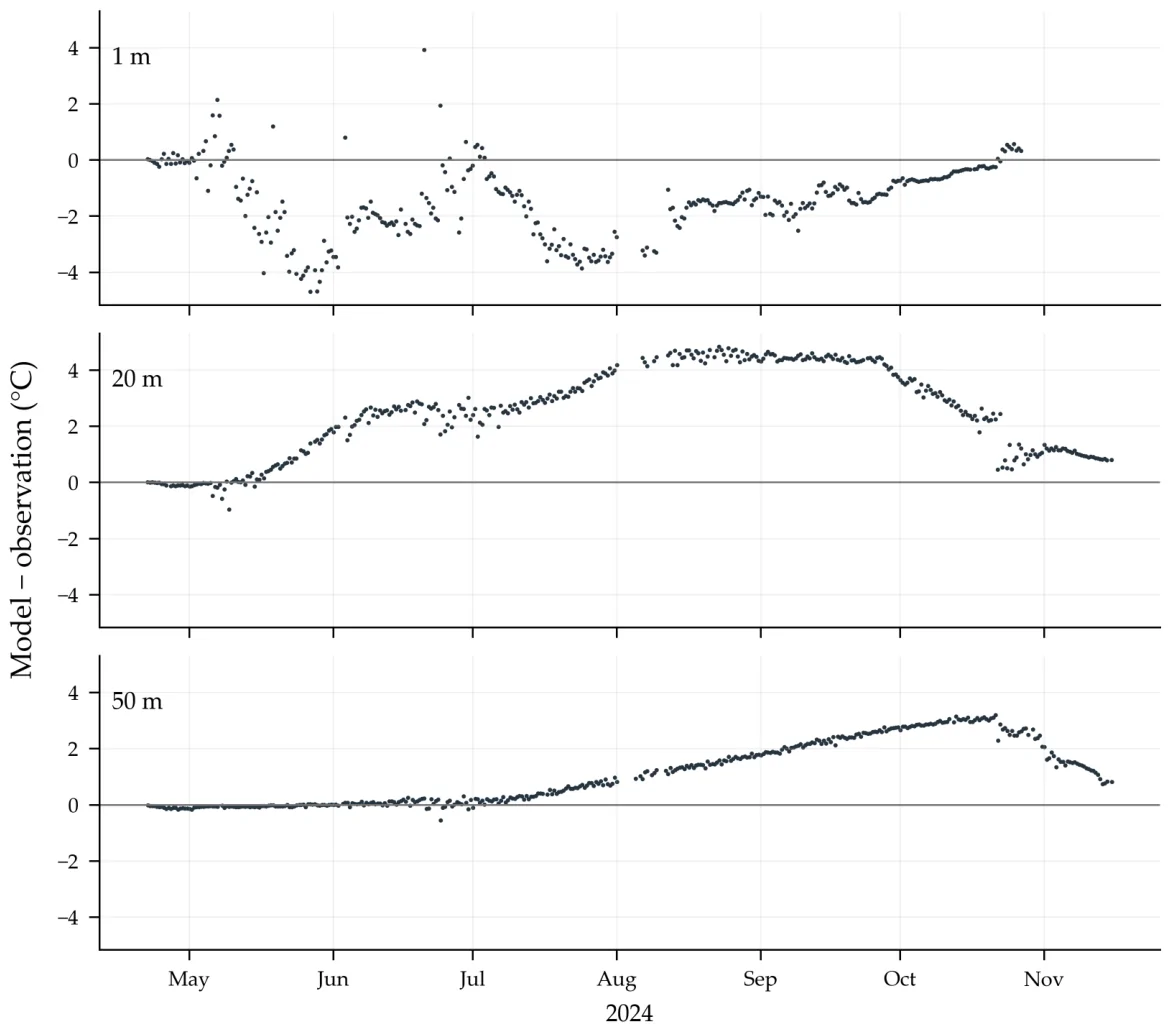
Temperature residuals for the thermal simulation at the profiler station, model minus observation.

## Data Availability

Model, scenario and sampling-analysis materials supporting the reported results are available at https://github.com/russellprimeau/source-water-digital-twin (accessed on 24 September 2026). Complete simulation reruns require licensed Delft3D FM and DELWAQ software and additional model inputs. Third-party lake-level and outflow records cannot be redistributed because of public-utility safety restrictions.

## Appendix A. Sensor Specifications and Calibration

This appendix summarizes the manufacturer specifications for the IoT sensing platforms described in Section 2.1, including calibration procedures and operational calibration history. Accurate calibration of these instruments is essential to the digital twin, since boundary condition data and model validation both depend directly on the reliability of the underlying sensor measurements. The instruments themselves are not a novel contribution of the paper. Both are commercially available products designed for scientific research, with documented sensitivity ranges, semi-automatic calibration and maintenance procedures available from the manufacturers online: profiler sonde (https://www.xylem.com/siteassets/brand/ysi/resources/manual/exo-user-manual-web.pdf, accessed on 24 September 2026) and the USV sonde (https://www.amloceanographic.com/product_documents/18, accessed on 24 September 2026).

These procedures are the basis for the operational calibration practices used in the course of collecting the data presented in the manuscript. Since it is stored between deployments, the sensors of the AML-3 sonde on board the USV are typically calibrated before each deployment. No AML-3 measurements are analysed in this manuscript, so its calibration records are not presented here.

For the profiler sonde, a history of calibration events relative to operational deployment is presented in Figure A1. Since procedures differ between sensors, to allow comparison between sensors with different numbers of calibration points, a linear function is fitted to represent each multi-point calibration event, as shown in Equation (A1):

(A1) y=Gx+O+e,

where *y* is the sensor output, *x* is the reference value, *G* is a fitted gain, *O* is a fitted offset, and e=y−(Gx+O) is the residual. For calibration events with too few points to estimate both gain and offset, *G* is fixed at 1 and only *O* is estimated; the residual is then the difference remaining after that offset correction.

Table A1 reports the measured parameters, the manufacturer accuracy and resolution specifications, and the operational calibration record for each channel. The fluorescent dissolved organic matter (fDOM) sensor carries no manufacturer accuracy specification; the manual states a linearity of $R^{2}>0.999$ over a serial dilution of a 300 ppb quinine sulfate standard and a detection limit of 0.07 ppb QSU instead. Calibration offsets summarize adjustments across events; their standard deviation is not the uncertainty of an individual environmental measurement. In particular, the turbidity offsets span a wide range relative to the lake’s low background signal, so this channel is not used as a quantitative model input. Manufacturer accuracy statements apply under their specified conditions and are distinct from field performance.

**Table A1:** Profiler sonde parameters, manufacturer specifications and calibration record.

Parameter	Units	Accuracy	Resolution	Cal. Pts	Cals.	Mean Offset	SD Offset
Temperature	°C	±0.01 from −5 to 35 °C; ±0.05 from 35 to 50 °C	0.001 °C	Factory calibrated; no field calibration			
Specific conductivity	μS/cm	±0.5% of reading or 1 μS/cm, whichever is greater	0.1–10 μS/cm	1	20	−1.61	21.25
Conductivity	μS/cm	Same sensor as specific conductivity, traceable to the same calibration					
Salinity	ppt	Derived from conductivity and temperature on the Practical Salinity Scale					
pH	pH	±0.1 within 10 °C of the calibration temperature; ±0.2 over the full range	0.01	3	21	−0.05	0.21
Dissolved oxygen	% sat.	±1% of reading or 1% air saturation, whichever is greater, to 200%; ±5% of reading from 200 and 500%	0.1%	1	25	−1.84	7.35
Turbidity	FNU	±2% of reading or 0.3 FNU, whichever is greater, to 999 FNU; ±5% from 1000 to 4000 FNU	0.01 FNU to 999 FNU; 0.1 FNU above	2	29	−8.88	43.11
fDOM	RFU	Not specified	Not separately specified	2	24	−0.11	0.11
fDOM	QSU	Not specified	0.01 ppb QSU	2	23	−0.62	0.52

## Appendix B. Model Configuration

The linear systems are solved with PETSc, the Portable, Extensible Toolkit for Scientific Computation, and the domain is decomposed across processes using MPI, the Message Passing Interface.

Table A2 records the configuration of the thermal simulation evaluated in Table 3 and Appendix C; Table A3 records the settings varied across the configurations compared in Figure 6. Neither describes the contaminant and sampling demonstrations.

**Table A2:** Delft3D FM configuration of the thermal simulation evaluated in Table 3 and Appendix C. It is neither one of the configurations compared in Figure 6 nor the scenario simulation.

Setting	Value
Solver and domain	
Computational kernel	D-Flow FM 1.2.184
Horizontal grid	Unstructured, 4935 faces in the thermal mesh
Vertical discretization	40 z-layers, 4 sigma layers at surface
Bed layering	Equal thickness in the lowest two layers
Bathymetry specification	At nodes; face levels from mean of node values
Latitude for Coriolis and heat flux	62.47° N
Matrix solver	Parallel PETSc
Domain decomposition	12 MPI partitions
Momentum advection	Perot, link-based
Vertical advection	Central implicit
Limiter, momentum and transport	Monotone central
Time	
Simulation period	22 April 2024 to 20 November 2024
Maximum timestep	20 s
Maximum Courant number	0.5
Forcing update interval	60 s
History output interval	3600 s
Map output interval	43,200 s
Turbulence closure	
Closure model	*k*-ϵ
$c_{1\epsilon }$	1.489
$c_{3\epsilon }$, stable stratification	0.0
$c_{3\epsilon }$, unstable stratification	1.489
Turbulent Schmidt number, salinity	0.7
Turbulent Prandtl number, temperature	0.193
Ozmidov length scale	0.027 m
Smagorinsky coefficient	0.2
Turbulence spin-up	43,200 s
Eddy viscosity and diffusivity	
Horizontal eddy viscosity $\nu_{H}$	$2.250\times 10^{-2}$ m^2^/s
Horizontal eddy diffusivity $D_{H}$	$9.533\times 10^{-2}$ m^2^/s
Vertical eddy viscosity $\nu_{V}$	$5.271\times 10^{-3}$ m^2^/s
Vertical eddy diffusivity $D_{V}$	$6.060\times 10^{-5}$ m^2^/s
Heat exchange and friction	
Heat flux model	Composite (ocean) model
Secchi depth	2.0 m
Stanton number, convective flux	−1.466 (as a multiple of wind drag)
Dalton number, evaporative flux	−0.794 (as a multiple of wind drag)
Albedo	0.06
Bed friction	Manning, 0.023
Wind drag breakpoints	0.00063 at 0 m/s; 0.00723 at 100 m/s
Initial and boundary conditions	
Initial water temperature	3.38 °C, uniform
Initial salinity	0.02 ppt, uniform
Inflows	Eight discrete tributaries, prescribed discharge
Outflows	Spjelkavikelva outlet and Vasstrandlia abstraction
Meteorological forcing	Weather station time series
Rainfall	Included

**Table A3:** Settings varied across the twenty-one configurations compared in Figure 6.

Setting	Range Covered
Horizontal mesh	2603 or 4935 two-dimensional cells
Vertical discretization	10, 20 or 40 z-layers
Water clarity	Secchi depth 0, 1, 2 or 7 m
Horizontal filtering	On or off
Vertical filtering	On or off
Vertical eddy viscosity	$10^{-6}$ to $10^{-3}$ m^2^/s
Vertical eddy diffusivity	$10^{-6}$ to $10^{-3}$ m^2^/s
Courant limit	0.4 or 0.7
Maximum timestep	15 or 30 s

These ranges describe the configurations compared in Figure 6. The configuration evaluated in Section 3.1 is reported in Table A2.

## Appendix C. Temperature Evaluation by Depth

Section 3.1 reports aggregate error statistics for the selected configuration, and Figure 5 plots all fifty depth groups at once. Because fifty overlapping traces obscure the behaviour of any one depth, this appendix isolates three from the same set of paired records.

Observations come from the profiler station, which records a vertical profile twice daily at approximately 1 m intervals. Model output is interpolated in time and depth to each observation, so that each comparison is made at the depth and time of the measurement rather than at the nearest model layer. Figure A2 shows 1, 20 and 50 m, representing the surface mixed layer, the transitional layer in which temperature falls sharply with depth, and the cold deep water beneath it.

The panels show that agreement depends on both depth and season. At 1 m the seasonal pattern is reproduced with Pearson correlation 0.959. At 20 m the simulated summer temperatures exceed the observations substantially. At 50 m the discrepancy grows through summer and early autumn before declining during cooling; it is not a constant offset. The residuals in Figure A3 show model minus observation at matched times and depths.

These patterns are consistent with excessive downward heat transport during stratification, but the temperature comparison alone does not isolate a particular mixing or surface-flux parameter as the cause.

Table A4 summarizes the error at 5 m intervals.

**Table A4:** Temperature error by depth for the thermal simulation, 2024 season.

Depth (m)	N	Pearson r	RMSE (°C)	MAE (°C)	Final Error (°C)
1	353	0.959	1.936	1.588	+0.323
5	351	0.965	1.315	1.096	+0.328
10	352	0.858	1.708	1.365	+0.337
15	352	0.774	2.753	2.306	+0.726
20	389	0.819	2.954	2.529	+0.804
25	385	0.842	2.721	2.290	+0.810
30	395	0.865	2.422	1.995	+0.810
35	396	0.876	2.158	1.731	+0.817
40	398	0.870	1.953	1.520	+0.833
45	398	0.870	1.754	1.312	+0.823
50	398	0.875	1.565	1.139	+0.818

The mean depth-band statistics summarize seasonal agreement; they are not bounds on peak errors or a guarantee of operational accuracy at a particular depth.

## Appendix D. Mass Balance of the Matched Conservative-Tracer Release

This appendix reports the whole-domain balance of the conservative tracer used in the transport-refinement study. It uses the same applied nitrogen mass, release cell, release hour and scenario discretization as Section 3.2, but it is a separate non-reacting calculation and does not test reaction accounting in the ammonium–nitrate simulation.

The solver writes a balance for each substance at every reporting interval: the total stock, the net contribution of reaction processes, the mass introduced by loads, and the mass crossing open boundaries in each direction. Conservation requires that the change in stock equals the sum of the flux terms; any discrepancy is mass created or destroyed numerically. Table A5 reports that balance for the matched conservative-tracer calculation, taken from the solver’s own balance output, not integrated afterwards from the concentration field.

**Table A5:** Whole-domain balance for the matched conservative-tracer release on the 4935-cell mesh with twenty layers, over the ten days following the release. Taken from the refinement study of Appendix E at the discretization the scenario simulation uses; this is not a balance of the reacting ammonium–nitrate calculation.

Term	Released Tracer (g N)
Initial stock in domain	0.0
Applied load	+3600.0
Boundary outflow	−148.0
Final stock in domain	3452.0
Residual (unaccounted)	0.05
Residual/applied load	14 ppm

The applied load in this matched calculation is 3600.0 g of nitrogen, and 95.9% of it remains in the domain ten days later, the rest having left through the outlet. The balance closes to 14 parts per million of the load. Table A7 reports the same conservative-tracer budget for all four discretizations of the refinement study, this one among them, which close to between 7 and 15 parts per million. These results demonstrate numerical conservation for the conservative transport calculation across both meshes and both vertical resolutions; they do not verify mass closure of the reacting ammonium–nitrate simulation.

Numerical diffusion spreads mass while conserving it, so where the material ends up is a separate question. Appendix E takes it up by refining the discretization.

## Appendix E. Transport Ensemble and Refinement of the Released Constituent

### Appendix E.1. Ensemble Membership

Seven hydrodynamic configurations from the comparison in Figure 6 were each used to carry one identical release: the same mass, at the same cell, over the same hour, as a conservative tracer with no reaction applied, for thirty days. All seven share the 4935-cell mesh, forty vertical layers and the same simulated period, and differ in the vertical eddy viscosity and diffusivity and in whether the vertical filter is active.

Membership was selected from thermal performance without reference to the tracer fields: a configuration is included if its seasonal temperature RMSE against the profiler record lies within a factor of two of the best among the seven. The comparison is confined to these seven because they share the mesh, the layer count and the simulated period, and an error accumulated over one window is not comparable with one accumulated over another. Table A6 records the seven and their RMSE. The five within 2.22 °C qualify; the two configured at $10^{-3}$ m^2^/s diffusivity fall outside and are not carried forward. The ensemble therefore contains five thermally plausible configurations under the stated selection rule; the factor-of-two threshold is a screening choice rather than evidence that their transport predictions are equivalent.

**Table A6:** Hydrodynamic configurations carrying the release, with temperature RMSE at the profiler station used to decide membership. All runs use the 4935-cell mesh with forty layers and cover 25 April to 20 November 2024; RMSE is evaluated against paired observations available through 15 November.

Configuration	Vertical Viscosity (m^2^/s)	Vertical Diffusivity (m^2^/s)	Vertical Filter	RMSE (°C)	In Ensemble
Vico6Dico5	$10^{-6}$	$10^{-5}$	on	1.110	yes
VicoDicoww5	$10^{-5}$	$10^{-5}$	on	1.110	yes
noVfilter	$10^{-6}$	$10^{-6}$	off	1.362	yes
Base	$10^{-6}$	$10^{-6}$	on	1.365	yes
Dicoww4	$10^{-6}$	$10^{-4}$	on	2.120	yes
VicoDicoww3	$10^{-3}$	$10^{-3}$	on	2.914	no
Dicoww3	$10^{-6}$	$10^{-3}$	on	2.916	no

### Appendix E.2. Refinement of the Released Constituent

Figure 6 records how the hydrodynamic solution responds to resolution. Whether the transport of a released constituent on that solution is resolved is a separate question, and it is the one the scenario results rest on. The release was therefore repeated on four couplings differing only in discretization, two horizontal meshes of 4935 and 2603 cells crossed with forty and twenty vertical layers, over the ten days following the release.

Fields on different meshes have no cell-by-cell correspondence, so the comparison is made on quantities that do not depend on the discretization: the depth-averaged concentration of the water column at four fixed geographic locations, and the whole-domain mass balance. The concentration in the surface layer is not such a quantity. That layer is twice as thick when the layer count is halved, so material remaining near the surface is averaged over twice the depth and the reported concentration falls by roughly half, for reasons that have nothing to do with the transport. Table A7 therefore reports depth averages.

**Table A7:** Peak depth-averaged concentration of the released tracer at four fixed locations, in μgN/m^3^, across two horizontal meshes and two vertical resolutions, with the whole-domain budget against the 3600.0 g applied. **Retained** is the share of that load still in the domain after ten days.

Cells	Layers	Source	Spjelkavikelva	Vasstrandlia	Profiler	Retained (%)	Residual (ppm)
4935	40	1857	2328	24.0	0.97	95.4	6.9
4935	20	1765	2117	26.3	0.97	95.9	13.8
2603	40	1544	2732	22.9	1.09	94.6	14.1
2603	20	1893	2230	26.2	1.10	95.3	14.9

Taking the four station peaks together, refining the horizontal mesh changes the depth-averaged concentration by a median of 6.3% at twenty layers and 14.8% at forty; refining the vertical resolution changes it by a median of 7.0% on the fine mesh and 16.4% on the coarse one. The largest single difference across the four comparisons is 22.6%. The same comparison made on surface-layer concentrations instead reaches 60.3%, the sampling-volume effect described above. The budget closes to between 7 and 15 parts per million of the applied load in every case, and between 94.6% and 95.9% of the release remains in the domain after ten days.

Across the four tested discretizations, the observed sensitivity of peak depth-averaged concentration has medians of 6 to 16% and a largest difference of 22.6%. These comparisons do not provide a grid-converged reference, establish monotonic convergence or bound the unknown discretization error. They quantify sensitivity over the tested meshes and layer counts and should not be interpreted as an accuracy margin for the reacting concentrations of Section 3.2.

## Appendix F. Operational Workflow, Data Coverage and Latency

### Appendix F.1. Implementation of the Update Loop

Table A8 describes how each stage of the loop in Figure 4 is implemented, and which stages run without intervention. The stages are those of a single update; the sequence has not been operated end to end as an unattended service, so the modes below describe how each stage runs rather than a demonstrated schedule.

**Table A8:** Implementation of each stage of the update loop.

Stage	Implementation	Mode
Model construction	Mesh, bathymetry and the initial parameter set are assembled by hand in the solver’s own tools and fixed before operation.	Manual
Sensor telemetry	Each platform is networked and publishes its current records as an HTML page served at its own address on the internet.	Continuous
Retrieval	A Windows service on a virtual machine runs a Python script on a fixed schedule, which fetches those pages, parses the tabulated records and applies range and plausibility checks.	Automated
Storage	Cleaned records are written to flat files, to a PostgreSQL database and to a version-controlled repository, so that no single store is the only copy.	Automated
Forcing assembly	Boundary and meteorological series are rebuilt from the stored records for the interval to be simulated, with substituted values tagged by origin.	Automated
Model execution	The solver runs as a separate application on a different machine from the acquisition host, launched for each run.	Manual
Evaluation and selection	Model output is scored against the profiler observations, which are withheld from the forcing, and the best-scoring member is kept.	Automated
Restart	The retained output supplies the initial condition for the following interval.	Automated
Publication	Selected output is displayed on the dashboard described in [8].	Manual
Vessel operation	Mission files are uploaded to the vessel, which is launched and recovered by hand, and the collected data are transferred after recovery.	Manual

The table reports component-level implementation rather than operation of a complete service. In particular, evaluation and restart scripts exist, but their use in a continuously scheduled ensemble update has not been demonstrated. Throughout this article, withheldmeans excluded from the model forcing and from any assimilation step; it does not mean withheld in time. The observations are contemporaneous with the interval being simulated, and no temporal hold-out is performed.

### Appendix F.2. Data Coverage

Table A9 reports the coverage of observations and meteorological forcing associated with the thermal simulation. It does not characterize the release scenario.

**Table A9:** Observation coverage for the thermal simulation, 22 April to 20 November 2024.

Stream	Coverage	Notes
Weather forcing	100%	Complete by construction: 94.0% from the on-site station, 6.0% backfilled from reanalysis and tagged as such
Profiler, surface sonde	88.5%	4504 of 5089 hourly intervals
Profiler, vertical profiles	95.8%	At least one profile on 204 of 213 days; 19,068 individual depth records
Water level	62.4%	3178 distinct hourly intervals; 3180 paired records

The meteorological dataset is complete after gap filling, with provenance tags identifying 94.0% station observations and 6.0% reanalysis. These percentages describe that dataset, not an operational retrieval success rate. Substituted values are drawn from the Norwegian Meteorological Institute’s 1 km Nordic analysis archive, sampled hourly at the grid cell nearest the station and covering the same parameters. Station observations are retained wherever they exist, and the analysis values enter unadjusted, without bias correction against the station record. Missing temperature observations are omitted from the pointwise comparisons; model values are interpolated to available observation times and depths.

### Appendix F.3. Computational Throughput and Latency

Nine retained runs completed 14 simulated days in a median of 32.5 min under twelve MPI partitions on the machine in Table 1. These runs share the horizontal mesh and the vertical discretization of the thermal simulation of Table A2 and cover a window inside its period, and differ from it only in their heat-exchange and turbulence coefficients, so the timing is representative of that configuration. This corresponds to approximately 620 simulated seconds per wall-clock second. It is a solver-throughput benchmark, not a measured duration for an hourly restart or an end-to-end operational update. It is also distinct from the serial model-configuration comparison in Figure 6.

Hourly acquisition makes near-real-time updates a plausible target, but total latency also includes retrieval, preparation, model execution, selection and publication. The full interval has not been measured. Twice-daily profiles additionally have a coarser observation cadence. The framework therefore does not claim a demonstrated under-two-hour publication latency.

### Appendix F.4. Robustness

Gap handling and data-quality processing are implemented components. End-to-end behaviour under retrieval failures, incomplete forcing and failed simulations has not been established through operational testing. Retention of the last successful output, explicit timestamps and stale-data alerts are requirements for a future unattended service, rather than demonstrated properties of this prototype.

## Appendix G. Stability of the Sampling Plan

The retained planning input comprises the 60 cells selected using the five ensemble members of Appendix E. Leave-one-cell-out and 2000 bootstrap replicates (random seed 20240401) repeat clustering and route selection. Geographical regions are identified by original cell membership, so DBSCAN label renumbering does not count as a different selected region. A bootstrap duplicate contributes to centroid and weight calculation, as in resampling the input rows.

Removing one cell at a time preserved the cluster count in 46 of 60 cases and the selected geographical regions in 53 cases. The baseline route travels 6942 m within the 7000 m budget. In 2000 bootstrap replicates, its selected region set recurred in 9.2% of cases. The most frequent region set was B–C–D–E, occurring in 26.2% of replicates.

Mean pairwise co-association within the two multi-cell baseline clusters ranged from 0.589 to 0.730, conditional on both cells being sampled. Singleton clusters have no within-cluster pair to assess. These summaries concern selected regions rather than exact centroid positions or visit order, both of which may change.

The two tests give different answers. Removing one cell at a time leaves the selected regions almost always unchanged, whereas bootstrap resampling, which perturbs the input far more, reproduces the exact set of regions only rarely, even though cells continue to fall together within the multi-cell clusters. These conditional tests show that the downstream plan is less sensitive to deleting one retained cell than to resampling the retained set, but they do not establish that the sensitive regions themselves are robust to alternative ensemble membership. The route travels 6942 m of a 7000 m budget, so a small change in weights makes a different combination affordable. The plan is one feasible option among several of similar value, not a uniquely optimal route.

Table A10 reports the same planning chain repeated across clustering neighborhoods. Between 40 and 300 m the summed score of the selected route varies by under 2% and its length by under 500 m, while the number of stations the mission must occupy falls from 19 to three. Over this tested range the neighborhood primarily controls grouping and station count, while the centroids and selected route can also change.

The results characterize the dependence of downstream planning on the retained cells, conditional on their scores and the fixed route budget. They are not confidence intervals for the concentration predictions. Resampling the cells is also a separate question from how the retained set responds to the membership of the ensemble that produced it, which is reported in Section 3.3 by recomputing the set from every subset of three and of four members.

**Table A10:** The sampling plan recomputed across clustering neighborhoods. Widest groupis the greatest distance between two cells placed in the same group, which the neighborhood does not bound. Stations is the number of centroids the selected route visits.

Neighborhood (m)	Groups	Stations	Widest Group (m)	Route Length (m)	Summed Score
40	21	19	331	6491	55.94
50	20	18	331	6489	55.94
60	16	14	331	6489	55.94
75	8	6	648	6484	55.94
100	6	5	648	6942	56.88
125	6	5	648	6942	56.88
150	4	3	1184	6872	56.94
200	4	3	1184	6872	56.94
250	4	3	1184	6872	56.94
300	4	3	1184	6872	56.94
